# Temporal phosphoproteomics reveals WEE1-dependent control of 53BP1 pathway

**DOI:** 10.1016/j.isci.2022.105806

**Published:** 2022-12-13

**Authors:** Valdemaras Petrosius, Jan Benada, Olaf Nielsen, Erwin M. Schoof, Claus Storgaard Sørensen

**Affiliations:** 1 Biotech Research and Innovation Centre (BRIC), University of Copenhagen, Ole Maaløes Vej 5, 2200 Copenhagen N, Denmark; 2 Cell Cycle and Genome Stability Group, Department of Molecular Biology, University of Copenhagen, Ole Maaløes Vej 5, 2200 Copenhagen K, Denmark; 3 Department of Biotechnology and Biomedicine, Technical University of Denmark, Søltofts Plads 224, 2800 Kgs. Lyngby, Denmark

**Keywords:** Molecular biology, Cancer, Omics

## Abstract

Wee1-like protein kinase (WEE1) restrains activities of cyclin-dependent kinases (CDKs) in S and G2 phase. Inhibition of WEE1 evokes drastic increase in CDK activity, which perturbs replication dynamics and compromises cell cycle checkpoints. Notably, WEE1 inhibitors such as adavosertib are tested in cancer treatment trials; however, WEE1-regulated phosphoproteomes and their dynamics have not been systematically investigated. In this study, we identified acute time-resolved alterations in the cellular phosphoproteome following WEE1 inhibition with adavosertib. These treatments acutely elevated CDK activities with distinct phosphorylation dynamics revealing more than 600 potential uncharacterized CDK sites. Moreover, we identified a major role for WEE1 in controlling CDK-dependent phosphorylation of multiple clustered sites in the key DNA repair factors MDC1, 53BP1, and RIF1. Functional analysis revealed that WEE1 fine-tunes CDK activities to permit recruitment of 53BP1 to chromatin. Thus, our findings uncover WEE1-controlled targets and pathways with translational potential for the clinical application of WEE1 inhibitors.

## Highlights

• We deciphered acute phosphoproteome alterations upon WEE1 kinase inhibition
• The changes mainly reflected activation of CDK1 and CDK2, but not other CDKs
• WEE1 indirectly suppresses phosphorylation of DNA repair and DNA replication factors
• WEE1 fine-tunes CDK activities to guide recruitment of 53BP1 to chromatin

## Graphical abstract

**Figure:**
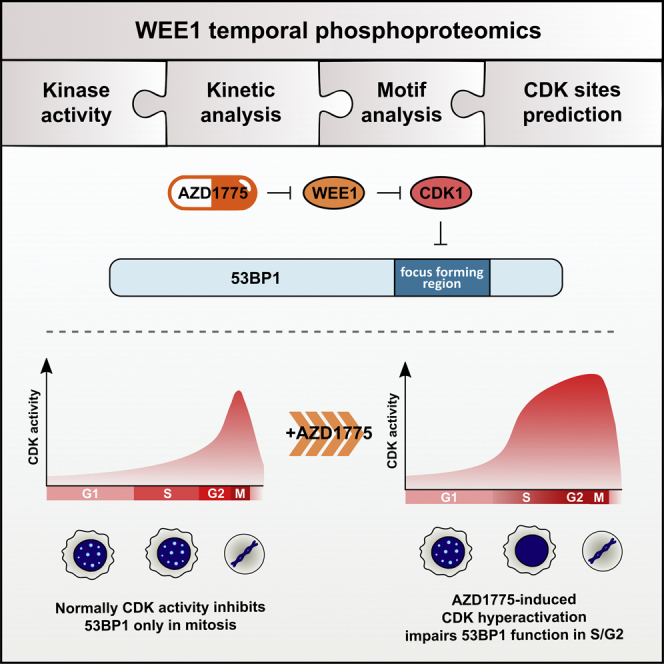

## Introduction

The eukaryotic cell cycle is orchestrated by specific cyclin-dependent kinases (CDKs) that mediate timely phosphorylation of key factors. As the process is unidirectional, cells employ cell cycle checkpoints to ensure the cellular demands are met before proceeding forward. Excessive genotoxic stress, as exemplified by the presence of double-strand breaks (DSBs), elicits cell cycle DNA damage checkpoints that delay progression via CDK inhibition. Accordingly, checkpoint activation provides time for repair, and in the case of DSBs, it is predominantly carried out by two repair pathways: classical non-homologs end joining (NHEJ) and homologous recombination (HR).^1^ 53BP1 and BRCA1 are two major DNA repair factors that play pivotal roles in regulating DNA repair pathway choice.^2^^,^^3^^,^^4^^,^^5^ 53BP1 promotes the NHEJ pathway with the help of its effector proteins such as RIF1.^3^^,^^6^ Notably, CDK activity also has to be tightly regulated during the normal cell cycle to ensure timely cell cycle progression as well as orderly execution of DNA replication.^7^^,^^8^^,^^9^ Thus, elevated aberrant CDK activity triggers illegitimate proliferation as well as genome instability.

The Wee1-like protein kinase (WEE1) governs a pathway that keeps CDK activity in check by inhibitory phosphorylation of Y15 on CDK1 and CDK2.^10^^,^^11^ WEE1 has a major role in promoting the G2/M checkpoint and controlling CDK1 activity; however, a paramount role for WEE1 in S phase via CDK2 has also emerged. Loss of WEE1 activity leads to massive origin firing, nucleotide depletion, MUS81/SLX4-mediated chromosome pulverization, and loss of replication fork protection.^8^^,^^12^^,^^13^^,^^14^^,^^15^

Cancer cells frequently display elevated expression of WEE1, likely to counterbalance the effects of oncogene activation or loss of p53 that otherwise trigger high levels of replication stress.^16^ WEE1 represents an attractive target for cancer therapy as WEE1 inhibition exploits cancer cell reliance on the G2/M checkpoint and exacerbates replication stress to intolerable levels. An inhibitor, adavosertib (AZD-1775), for WEE1 has been developed^17^ and it has been the focal point of 60 clinical studies (clinicaltrials.gov, 2021-11). Although, most studies aim to specifically exploit the role of WEE1 in the G2/M checkpoint, evidence has emerged that the S phase impacts of WEE1 inhibition contribute to cancer-specific lethality.^18^^,^^19^^,^^20^ Furthermore, positive outcomes were reported from phase II clinical trials for pancreatic and ovarian cancer by combining adavosertib and gemcitabine treatment, exploiting high levels of replication stress in these types of cancers.^21^^,^^22^ Considering the biological importance of WEE1 as well as the potential for wide use of adavosertib or other WEE1 inhibitors in clinical settings, it is surprising that comprehensive WEE1-focused phosphoproteomes have not been reported. Here, we systematically investigated the WEE1-regulated phosphorylation dynamics by utilizing quantitative time-resolved phosphoproteomics to explore signaling cascades that respond to WEE1 inhibition. Through tailored bioinformatic analysis, we expanded on the current knowledge of proline-directed S/T kinases and kinome-wide effects of WEE1. Furthermore, we uncovered that inhibition of WEE1 led to hyperphosphorylation and inactivation of key DNA repair proteins. We identified WEE1-regulated targets and kinase pathways that can provide valuable information for the design of combinatorial therapeutic strategies with WEE1 inhibitors.

## Results

### Phosphoproteomic analysis of adavosertib-treated samples

**Figure 1:**
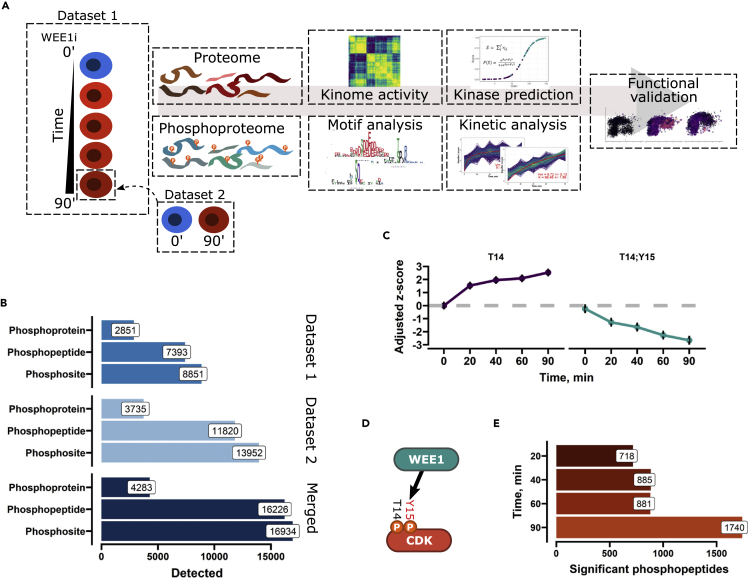
WEE1 inhibition leads to rapid large-scale alterations in the phosphoproteome (A) Experimental and computational workflow overview. Two datasets are generated, a time-resolved (20, 40, 60, 90 min n = 3) and single time point (90 min n = 4).WEE1i indicates adavosertib treatment, and n the number of replicates in each dataset. (B) Bar plot of detection summary of phosphoproteomic datasets. Number phosphoproteins, phosphopeptides, and phosphorylation sites for individual and combined datasets are shown. (C) Plot of CDK inhibitory t-loop T14 and Y15 phosphorylation quantification Z-scores are adjusted so that the first time point is equal to zero. Black bars represent the error on the adjusted *Z* score. (D) Illustration of T-loop inhibitory phosphorylation of CDK1 and CDK2 by WEE1. (E) Significantly altered phosphopeptides at different time points were determined by statistical testing. False discovery rate (FDR) < 0.05.

WEE1 inhibition has acute effect leading to CDK hyperactivation and phenotype impacts within the first hour of treatment.^8^^,^^17^ Thus, to study the role of WEE1 through phosphoproteome analysis, cells were incubated with adavosertib for 20, 40, 60, and 90 min or only at a single time point of 90 min generating two separate datasets that were integrated to achieve higher phosphoproteome coverage (Figure 1A). The sarcoma cell lines U2-OS was selected for the proteome studies as it has been well characterized for WEE1 functions, notably, adavosertib and more prolonged WEE1 siRNA treatment yielded comparable results.^8^ To elicit a strong burst in CDK activity while minimizing effects on cell cycle position, we treated the cells 1 μM of adavosertib which has been shown to systematically increase CDK substrate phosphorylation and almost completely abolish inhibitory T-loop phosphorylation on CDK1 within 60 min,^8^ albeit such a higher concentration might affect the activity of other kinases. Notably, the 1 μM concentration corresponds in the order of magnitude to the plasma levels of adavosertib in patients treated with 175 mg of the drug, a commonly used dose in clinical trials.^23^ We did not extend treatment beyond the 90 min time point as cells accumulated DNA damage which could convolute our results due to the secondary cellular events (Figure S1A). The global scale of protein phosphorylation was quantified by liquid chromatography-tandem mass spectrometry (LC-MS/MS) in combination with tandem mass tag labeling. Ti-IMAC was used for the enrichment of phosphorylated peptides. The quantified protein and phosphopeptide abundances were normalized with a custom algorithm (see STAR Methods) and principal component analysis was used to assess the technical quality of the data (Figures S1B and S1C). The samples treated with adavosertib clearly separated from control in the first principal component indicating good reproducibility. We quantified >16,000 phosphosites with no missing values from >4,000 phosphoproteins (Figure 1B). We observed a linear decrease in the abundance of the phosphopeptides carrying the double inhibitory T14:Y15 phosphorylation, and a corresponding increase in the single T14 (Figure 1C). CDK1 and CDK2 yield an identical tryptic peptide containing the T14; Y15 modifications, so we could not discern specific changes for these two kinases. However, based on previous work,^10^^,^^11^ we assumed that phosphorylation decreases on both kinases. We did not obtain any other direct evidence that would indicate that WEE1 can phosphorylate other targets apart from CDK1 and CDK2. The decrease in the inhibitory Y15 phosphorylation was accompanied by increased CDK substrate phosphorylation (Figure S1D), indicating suppressed WEE1 function and subsequent activation of CDKs. After statistical testing, we detected an increasing number of significantly altered phosphopeptides throughout the timecourse after correction for changes in total protein abundance. Already after 20 min of treatment, we detected 718 differential phosphopeptides and 1,704 at the final time point (Figure 1E). Inhibition of WEE1 had little impact on asynchronous cell cycle distribution (Figure S1E), though we did observe a gradual CDK-dependent increase in the population of mitotic cells (Figures S1F and S1G). Taken together, we generated data to study immediate adavosertib impacts, which includes the WEE1-CDK signaling axis as well as global effects on phosphorylation dynamics.

### Adavosertib treatment leads to a rapid increase in CDK1 and CDK2 activity

**Figure 2:**
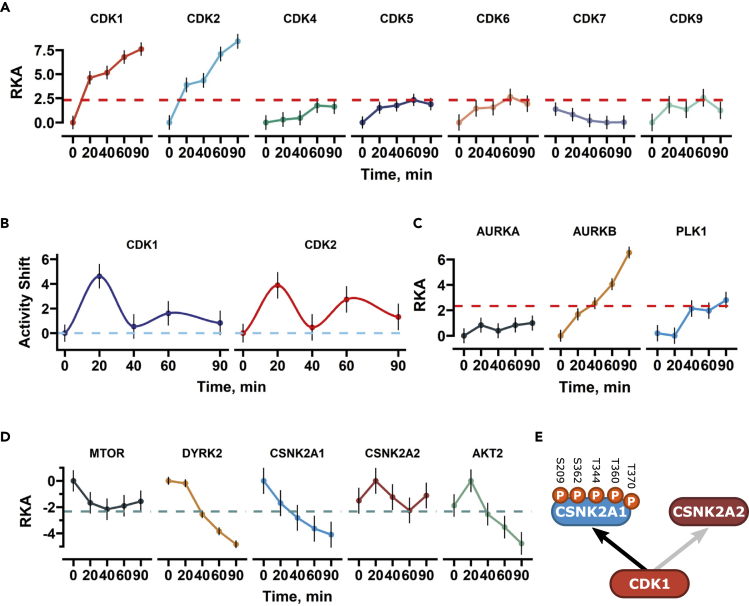
Rapid kinase activity impacts of adavosertib extend beyond CDK1 and CDK2 (A) Relative kinase activities (RKA) of quantifiable CDKs, that have >5 detected phosphorylation sites. The red bar indicates the significance (p = 0.01) boundary. Black bars indicate the SE of the activity score. (B) Relative kinase activity shifts for CDK1 and CDK2. Black bars indicate the propagated SE of the activity shift. (C and D) RKA plot showing activity of other affected kinases that were correlated with CDK activity. (E) Illustration of CK2 subunit CSNK2A1 and CSNK2A2 known CDK-dependent phosphorylation sites.

Alterations in kinase activities or their downstream pathways underlie cancer sensitivity to specific kinase inhibitors as well as to other chemotherapeutics.^24^ To better understand the kinome-wide effects of WEE1 inhibition, we inferred relative kinase activities by modifying a previously used approach.^24^ Detected phosphosites were annotated with respective kinases from the Omnipath database,^25^ and Stouffer’s *Z* score combination method was used to combine standardized phosphorylation site abundances into a single value for each time point (see STAR Methods). To avoid faulty activity estimates, only the activity of kinases with at least 5 quantified sites in our datasets was considered. To determine if adavosertib via WEE1 may regulate other CDKs, apart from CDK1 and CDK2, we first investigated the relative kinase activities (RKA) for all possible CDKs. Inhibition of WEE1 led to the increased relative activities of CDK1 and CDK2, but did not markedly affect other CDKs (Figure 2A), which corroborates original research carried out on the activity of WEE1.^10^^,^^11^ Significant activation for CDK6 and CDK5 was observed at later time points; however, this was rather minor compared to CDK1 and CDK2. The largest increase in activity of CDK1 and CDK2 was observed after 20 min and continued increasing albeit more modestly (Figure 2B). This suggested that only CDK1 and CDK2 responded rapidly to the loss of WEE1 function.

### WEE1 affects multiple kinase pathways

We reasoned that multiple kinases might be affected by adavosertib; thus, we utilized the time-resolved nature of our dataset and performed activity correlation analysis based on relative kinase activity (Figure S2). From the correlation map, we detected positive kinase activity correlations between other known cell cycle driving kinases such as aurora kinases B (AURKB) and the polo-like kinase 1 (PLK1), recapitulating expected activation of mitotic kinases. The AURKB kinase activity increased linearly throughout the timecourse, while PLK1 was activated only in the later time points (Figure 2C). It has been noted that adavosertib can suppress the activity of PLK1 *in vitro*;^26^^,^^27^ however, further research showed that adavosertib treatment stimulates PLK1 activity *in cellulo*,^28^in line with our findings. In contrast, we observed a negative RKA correlation with kinases such as mammalian target of rapamycin (mTOR), casein kinase 2 (CK2) dual-specificity tyrosine phosphorylation-regulated kinase 2 (DYRK2), and AKT (Figure 2D), which might reflect off-target effects of adavosertib at the applied concentration. The available binding-based kinome profiling for the compound might be used to rule out these effects;^26^ however, it was shown that adavosertib binding is a poor proxy for activity effects.^27^ Generally, it is desirable to avoid off-target effects; however, considering the high concentrations used in the clinical settings (175 mg doses approximating 1 μM in plasma), the identified kinase activity changes are also potentially present in patients. Despite these limitations, the CK2 kinase could be a focus for further mechanistic studies. CK2 controls vital cellular pathways and can contain two catalytic subunits: CSNK2A1 and CSNK2A2.^29^ While CSNK2A1 activity linearly decreased, CSNK2A2 showed no clear trend suggesting that adavosertib-driven high CDK activity only affects one of the catalytic subunits (Figure 2D). In line with this, there are five CDK sites present on CSNK2A1, in contrast to CSNK2A2 which is devoid of them (Figure 2E). Previous studies found that adavosertib does not affect CSNK2A1 kinase activity *in vitro* at the same concentration as used in our assay,^27^ indicating that WEE1 can potentially affect CK2 function by suppressing CDK activity.

### Phosphorylation profile analysis

Phosphorylation events are highly dynamic being influenced by altered activity of multiple kinases and phosphatases or other cellular processes; hence, protein phosphorylation status might differ at distinct time points during a time course. For this reason, studying phosphorylation dynamics can provide valuable information that has important functional implications. To identify patterns of phosphorylation dynamics, we utilized fuzzy c-means clustering, which has been shown to perform well for time-resolved phosphoproteomic datasets.^30^ We classified the phosphorylation sites into eight clusters based on their phosphorylation patterns by using calculated optimal parameters. Clusters 1, 4, and 6 showed only transient changes in phosphorylation levels after 20 min, while cluster 3 exhibited a delayed increase in phosphorylation levels (Figure S3A). Furthermore, statistically significant alterations in phosphorylation levels in clusters 1 and 4 were highest after 20 min of WEE1 inhibition, suggesting potentially important signaling events even in this early time point (Figure S3B).

**Figure 3:**
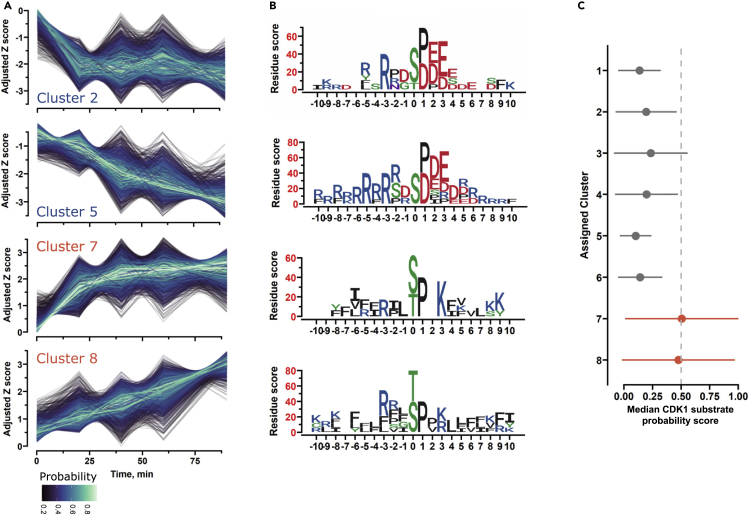
Phosphorylation dynamic analysis reveals potential CDK-driven events triggered by WEE1 inhibition (A) Fuzzy-*c*-means (FCM) cluster profiles for select clusters, time is plotted on the x axis and adjusted Z scores on the y axis. Each line corresponds to the dynamics of a single phosphopeptide. Color intensity represents the probability of classification to the respective cluster. Z-scores are adjusted by adding or subtracting the minimal value. (B) Phosphorylation sites sequence motifs for the clusters in A generated with a binomial probability model. x axis indicates the amino acid position from the phosphorylation site. Amino acid colors are based on chemical properties. (C) Median CDK1 substrate probability scores are based on position-specific scoring matrixes (PSSM). Clusters 7 and 8 that may capture CDK-dependent events are indicated in red. The line indicates the median absolution deviation of the median CDK1 substrate probability score.

Four clusters (2, 5, 7, and 8) showed a stable change in phosphorylation levels, with a trend that was either linear or the relative phosphorylation levels quickly shifted at 20 min and remained elevated afterward (Figure 3A). Sites stratified to clusters 2 and 5 displayed an overall decrease in relative abundance. Notably, they exhibited a motif that is characteristic of CSNK2A1 (Figure 3B) which is in accordance with the previously indicated decreasing CSNK2A1 activity (Figure 2D). In contrast, clusters 7 and 8 displayed increasing levels (Figure 3A). Motifs generated for these clusters had enriched residues that markedly resembled the consensus sequence (S/T-P-x-K/R) for CDK target sites (Figure 3B), suggesting that these clusters predominantly contained substrates for these kinases. To numerically quantify the clustered phosphosite similarity to known CDK substrates, we built position-specific scoring matrixes (PSSM).^31^^,^^32^ To generate the CDK1- and CDK2-specific PSSMs, we extracted phosphorylation sites with adjacent amino acids sequences from the PhosphositePlus and SIGNOR databases via Omnipath and calculated the amino acid position scores with a binomial probability model^31^ with disorder-adjusted background frequencies (Figure S3C). We then carried out scoring for the detected phosphorylation sites and transformed the arbitrary scores into probabilities with logistic regression. In accordance with the generated motifs, we observed the highest scores in clusters 7 and 8 (Figure 3C). Based on the collective data and analysis, we suggest that clusters 7 and 8 contain CDK-dependent events downstream of WEE1 inhibition.

### Empirical prediction of CDK substrates

**Figure 4:**
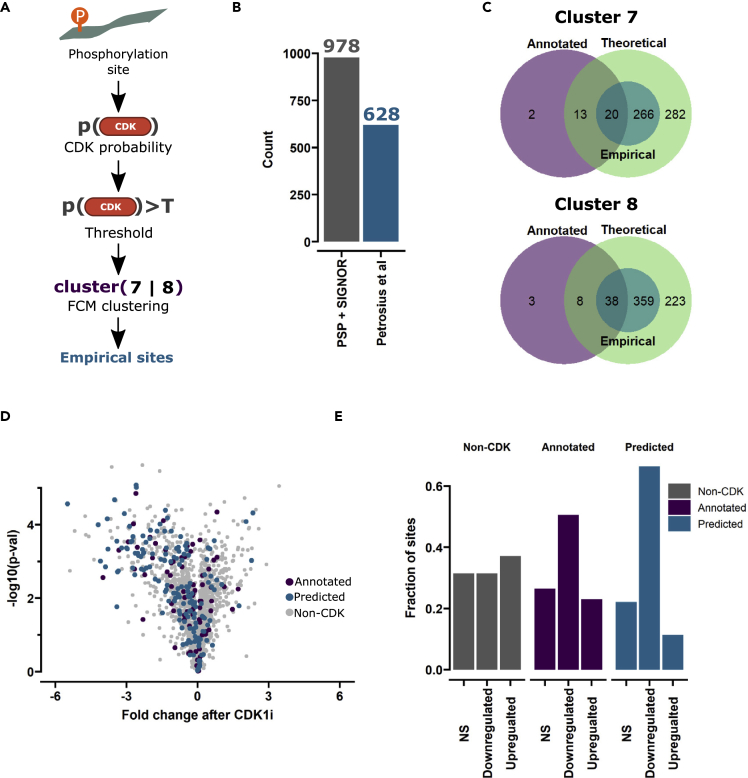
Prediction of large number of CDK sites through time-resolved phosphoproteomic data in combination with previous knowledge databases (A) Scheme for prediction of theoretical and empirical CDK sites. A probability for CDK1 or CDK2 is calculated for every quantified phosphorylation site, which is used to classify the sites into non-CDK and theoretical CDK sites. Experimental data are then taken into account, by further filtering the potential CDK list for FCM stratification and significant upregulation. (B) Bar plot showing annotated CDK sites in PhosphoSitePlus (PSP) and SIGNOR databases and the number of predicted sites in this study (Petrosius et al.). (C) Venn diagram showing the overlap between annotated (PSP and SIGNOR databases), theoretical (highest PSSM probability score for CDK1 or CDK2), and empirical CDK sites. (D) Volcano plot, based on quantification data obtained from Petrone et al., 2016. The fold change is plotted on the x axis and the negative log10 of the p value on the y axis. Predicted and annotated CDK sites are marked with distinct colors. (E) Bar plot showing fractions of non-CDK, annotated CDK, and predicted CDK sites that are not significantly altered (NS), upregulated, or downregulated after CDK1 inhibition.

We set out to determine if phosphosites in clusters 7 and 8 were previously identified and assigned to CDKs. However, only a small fraction of the detected phosphorylation sites could be linked with CDK1 and CDK2 kinases based on PhosphositePlus and SIGNOR databases (Figure S4A). Lack of phosphosite annotation can indicate an incomplete view of the signaling cascades affected by WEE1. To remedy this, we utilized our PSSM approach to identify potential CDK1 and CDK2 target sites concurrently deconvoluting hyperphosphorylation events dependent on other kinases. To evaluate the ability of our method to accurately predict already annotated CDK cites, we carried out receiver-operating characteristic (ROC) analysis, which demonstrated that the PSSM prediction algorithm could accurately distinguish CDK substrates (Figure S4B) with AUC values of 0.943 (CDK1) and 0.882 (CDK2). From the ROC analysis, we obtained CDK probability cutoff values that were used to classify the phosphorylation sites as CDK1 and CDK2 targets. Categorization of CDK-specific substrates is challenging due to similar consensus motifs and overlap between targets. In the absence of CDK2, CDK1 complements its role in driving the cell cycle,^33^ further underlining the high degree of similarity. For this reason, we combined the predicted sites from CDK1 and CDK2 into a common list of potential CDK sites. By ranking kinases based on predicted probability, we identified 798 potential CDK sites. We performed gene set overrepresentation analysis for these sites and found that they were primarily found on proteins involved in cell cycle-related processes, the expected biological themes for CDK substrates (Figure S4C). To improve the accuracy of our identified CDK sites, we integrated the theoretical prediction with our empirical data. We assumed that CDK sites would exhibit increasing phosphorylation levels after WEE1 inhibition due to elevated CDK activity (Figure 2A). Phosphorylation sites that were classified to either cluster 7 or 8 and had significantly increased (FDR <0.05) phosphorylation levels were further filtered based on their CDK probability with cutoff scores obtained from ROC analysis (Figure 4A). This approach generated a list of 628 tentative new CDK sites, rivaling the number of currently known sites in databases (Figure 4B). The identified sites also had a good overlap with already known CDK phosphorylation sites. In cluster 8, the vast majority of predicted sites overlapped with annotated CDK sites and more than half of them overlapped in cluster 7 (Figure 4C). However, multiple annotated CDK sites were not classified accordingly with our empirical approach.

To substantiate the validity of our identification, we compared the predicted sites with an independent study that used chemical inhibition of CDK1 with 5 μM RO-3306 to identify potential CDK1 sites.^34^ We extracted the abundance quantification of sites detected in our dataset and categorized them based on the fold change and p value into unaltered, upregulated, and downregulated (Figures 4D and 4E). The non-CDK sites were equally distributed in all three categories, while known CDK sites had a higher fraction of downregulated sites. Notably, our identified sites exhibited an even more evident enrichment of downregulated sites after CDK1 inhibition, supporting the reliability of our prediction (Figure 4E). To more directly confirm our predicted CDK sites, we co-treated cells with both adavosertib and the CDK1 inhibitor RO-3306 that also was used in the Petrone et al. study.^34^ Globally, the predicted CDK sites displayed increased phosphorylation after WEE1 inhibition, which was reduced upon addition of the CDK1 inhibitor (Figure S4D), further corroborating our prediction accuracy claims. Overall, our identified phosphorylation sites can markedly bolster the list of database-contained CDK sites and serve as a valuable resource.

### CDK substrates are phosphorylated with distinct dynamics

**Figure 5:**
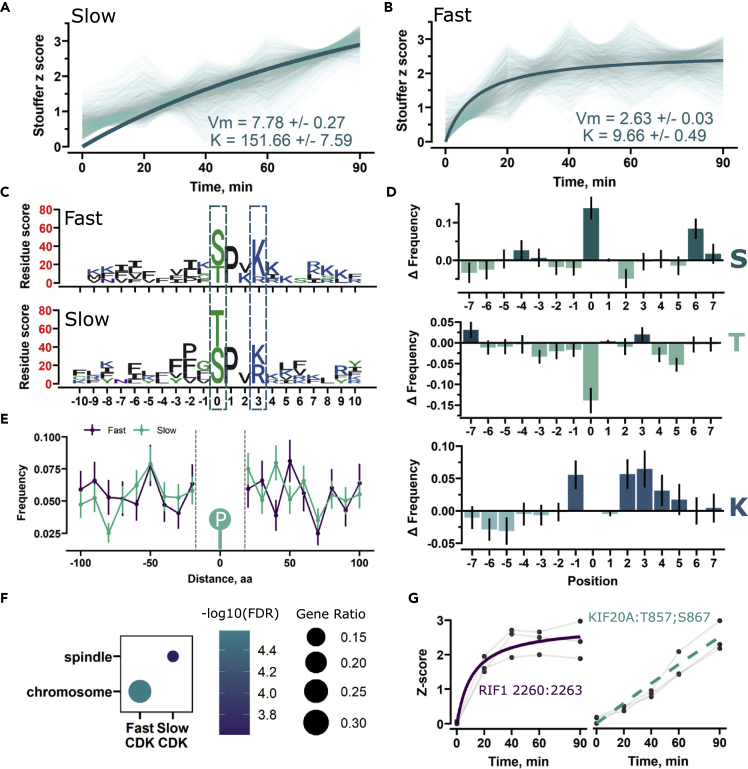
Putative CDK substrates display bimodal phosphorylation dynamics after WEE1 inhibition (A and B) Analysis of clusters 7 and 8 from FCM clustering with Michaelis-Menten modeling. Faded lines represent phosphorylations sites. The model parameters Vm and K are indicated in the plot. (C) Sequence motif generated for predicted and annotated CDK sites stratified to either the slow or fast cluster. Coloring is based on amino acid chemical properties. (D) Frequency differences for serine (S), threonine (T), and lysine (K) residues between fast and slow clusters. The amino acid position is plotted on the x axis and the frequency different on the y axis. The black bars represent the SD of the frequency estimated by resampling. (E) Potential Cy motif frequency at different binned distances from the phosphorylation site. Bars indicate the SD of the frequency estimated by resampling. (F) Cellular compartment comparison for CDK sites in slow and fast clusters. (G) Phosphorylation trend of top scoring phosphorylation sites in the fast and slow clusters. Points represent the standardized abundances, which are connected by faded lines to represent the distinct replicates. The Michaelis-Menten fit is shown in the plot. In the case of KIF20A, the fit did not converge, so a straight line is used instead.

We were surprised that phosphopeptides in both clusters 7 and 8 had similar motifs and CDK probability scores, even though they appeared to exhibit distinct phosphorylation dynamic patterns (Figures 3A and 3B). To directly compare the two clusters, we performed Michaelis-Menten modeling on these clusters to statistically assess the difference between them. Based on the model, cluster 7 had a much slower phosphorylation half-time (K) compared to cluster 8. However, the phosphosites classified to cluster 8 could get phosphorylated to a higher extent, suggesting the presence of two distinct phosphorylation patterns for CDK substrates (Figures 5A and 5B). Based on this, the clusters were referred to as “slow” and “fast”.

To characterize this “slow” and “fast” dichotomy, we extracted the predicted and known CDK sites found in these clusters and analyzed different features selected based on known CDK specificity determinants that might be affecting the phosphorylation rate.^35^^,^^36^^,^^37^ We generated cluster-specific sequence motifs to pinpoint potential differences in the phosphorylation motif. The motifs revealed a notable difference in the preferred phosphorylation residue and the +3 position, where the lysine residue had a higher score in the fast cluster compared to the slow (Figure 5C). To directly compare any significant frequency differences, we resampled the motifs 100 times to estimate the mean and uncertainty of serine, threonine, and lysine residues up to seven amino acids from the phosphorylation site (Figure 5D). Phosphorylation sites stratified to the fast cluster displayed an increased frequency of serine residues with a corresponding decrease in threonine. In comparison to the optimal CDK consensus motif (S/T-P-x-K), we observed an increased frequency of lysine residues at position +3. Additionally, lysine residues were more frequent at position +2 and +4 (Figure 5B). Although these residues are not part of the canonical motif, they have been shown to act as determinants of CDK substrate specificity.^38^ Together, these difference in the phosphorylation motifs contribute to more rapid phosphorylation.^39^

CDKs are recruited to their substrates with the help of cyclins, which recognize cyclin-binding (Cy) motifs on the target protein. In yeast, it has been shown that functional Cy motifs need to be within 20–80 amino acids from the phosphorylation site to function and can play an important role in regulating substrate phosphorylation timing.^39^^,^^40^ Thus, we extracted the potential Cy motifs on both sides of known CDK sites with the use of a regular expression. However, we did not observe any major difference in the availability of potential Cy motifs surrounding the phosphorylation sites (Figure 5E), though detection of distinct differences might be obscured due to the use of regular expressions, as the amino acid match alone does not guarantee functionality.

Finally, we performed gene set overrepresentation to investigate if there were significant differences in cellular compartmentalization between the clusters. The quickly phosphorylated sites were enriched for chromosomal association, albeit the slow sites did not exhibit the same enrichment, suggesting that sites found on proteins with specific cellular localization are more likely to exhibit fast phosphorylation dynamics (Figure 5F). As example case, we extracted the phosphorylation sites of RIF1 and KIF20A that are fast and slow, respectively (Figure 5G). As expected, we observed a striking difference in the phosphorylation profile of these sites. From these observations, we concluded that the phosphorylation dynamics of CDK phosphorylation sites was impacted by both substrate cellular localization and intrinsic motif properties.

### Adavosertib triggers hyperphosphorylation of key DNA replication and repair factors

**Figure 6:**
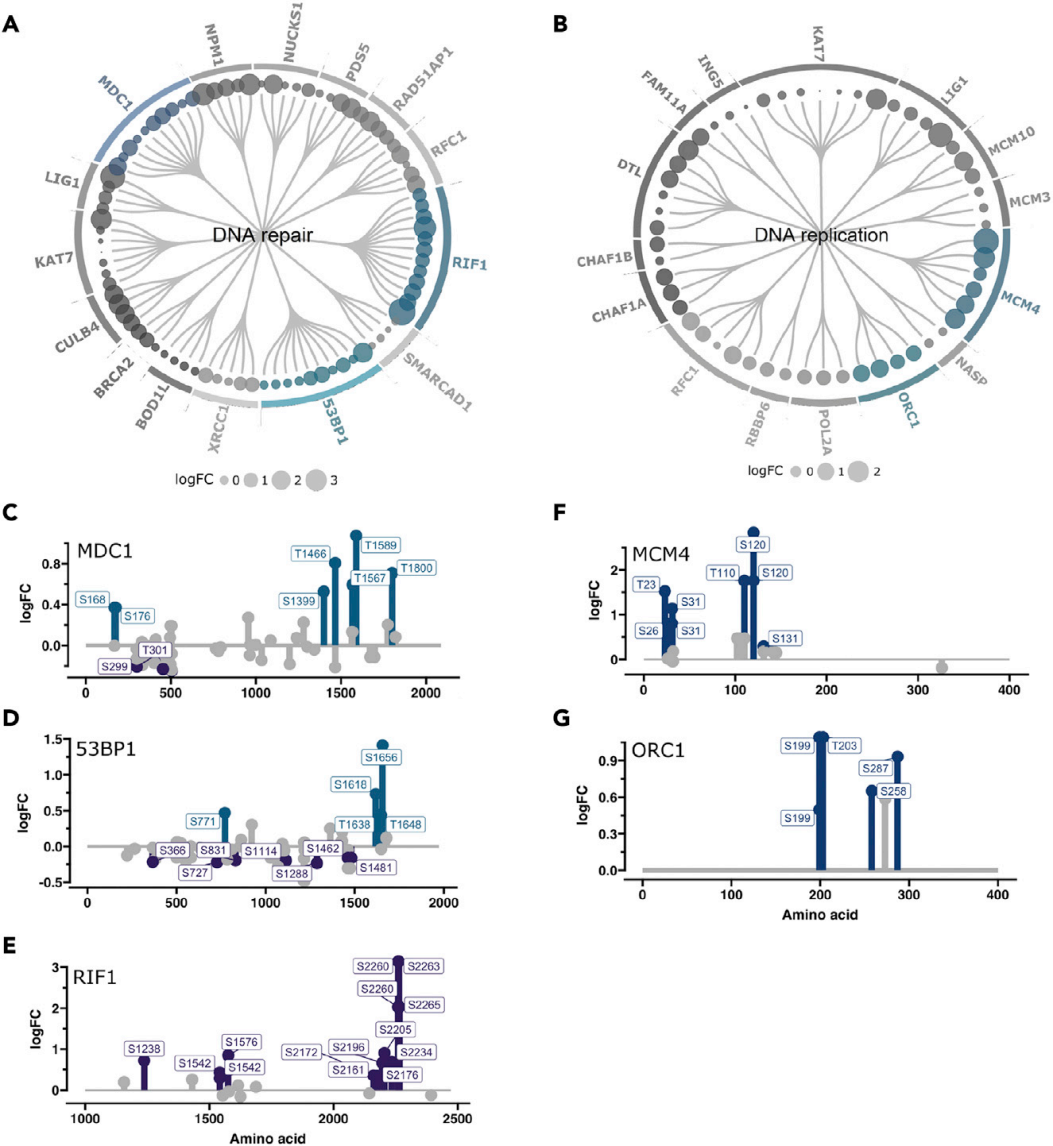
Adavosertib triggers phosphorylation of multiple clustered sites in DNA replication and repair factors Dendrogram visualizing detected significantly (FDR <0.01) altered phosphopeptides for proteins involved in DNA repair (A) and replication. (B) Dot size indicates the logFC after 90 min of WEE1 inhibition. Plots showing the phosphorylation pattern of (C) MDC1, (D) 53BP1, (E) RIF1, (F) MCM4, and (G) ORC1. The logFC (90 min) of a phosphosite is indicated on the y axis and the amino acid sequence on the x axis. Significantly altered phosphorylation sites are marked with a label indicating the phosphorylated residue position on the protein. Sites are merged from datasets 1 and 2.

The phosphoproteomic datasets are natural starting points for hypothesis-based exploration. Interestingly, after 20 min of adavosertib treatment, phosphorylation sites on RIF1 (S2260:S2263) and MCM4 (S120) proteins have the highest response. The two proteins play vital roles in DNA replication and DNA repair.^3^^,^^6^^,^^41^^,^^42^^,^^43^^,^^44^ As these processes are highly relevant for cancer therapies, we investigated if additional proteins within the pathways displayed hyperphosphorylated sites. We extracted all proteins that were associated with DNA replication and repair gene ontologies from our dataset and plotted the proteins with the largest number of significant sites into a circular dendrogram, where each node corresponds to a significantly altered phosphopeptide (Figures 6A and 6B). WEE1 inhibition led to hyperphosphorylation of multiple residues in DNA replication factors, such as LIG1 and ORC1 (Figure 6B). Furthermore, we observed increased phosphorylation of several DNA repair proteins such as BRCA2, RAD51AP1, and XRCC1. Notably, we observed an especially large number of phosphosites on MDC1, 53BP1, and RIF1 (Figure 6A). To investigate if these sites were situated in known functional regions, we plotted the phosphorylation profiles for these factors. We noted that the sites were primarily localized to the far C-terminal region for all three DNA repair factors (Figures 6C–6E). Hyperphosphorylated sites on MCM4 and ORC1 were in the N-terminus and the central part, respectively (Figures 6F and 6G). It has been noted that phosphorylation of the C-terminal region of 53BP1 and RIF1 can inhibit their function.^45^^,^^46^^,^^47^^,^^48^ Moreover, phosphorylation sites with the largest effect sizes on 53BP1 and RIF1 were predicted as putative CDK sites based on our model and exhibited fast dynamics stratifying them to the “fast” FCM cluster.

### WEE1 promotes 53BP1 function by limiting CDK activity

**Figure 7:**
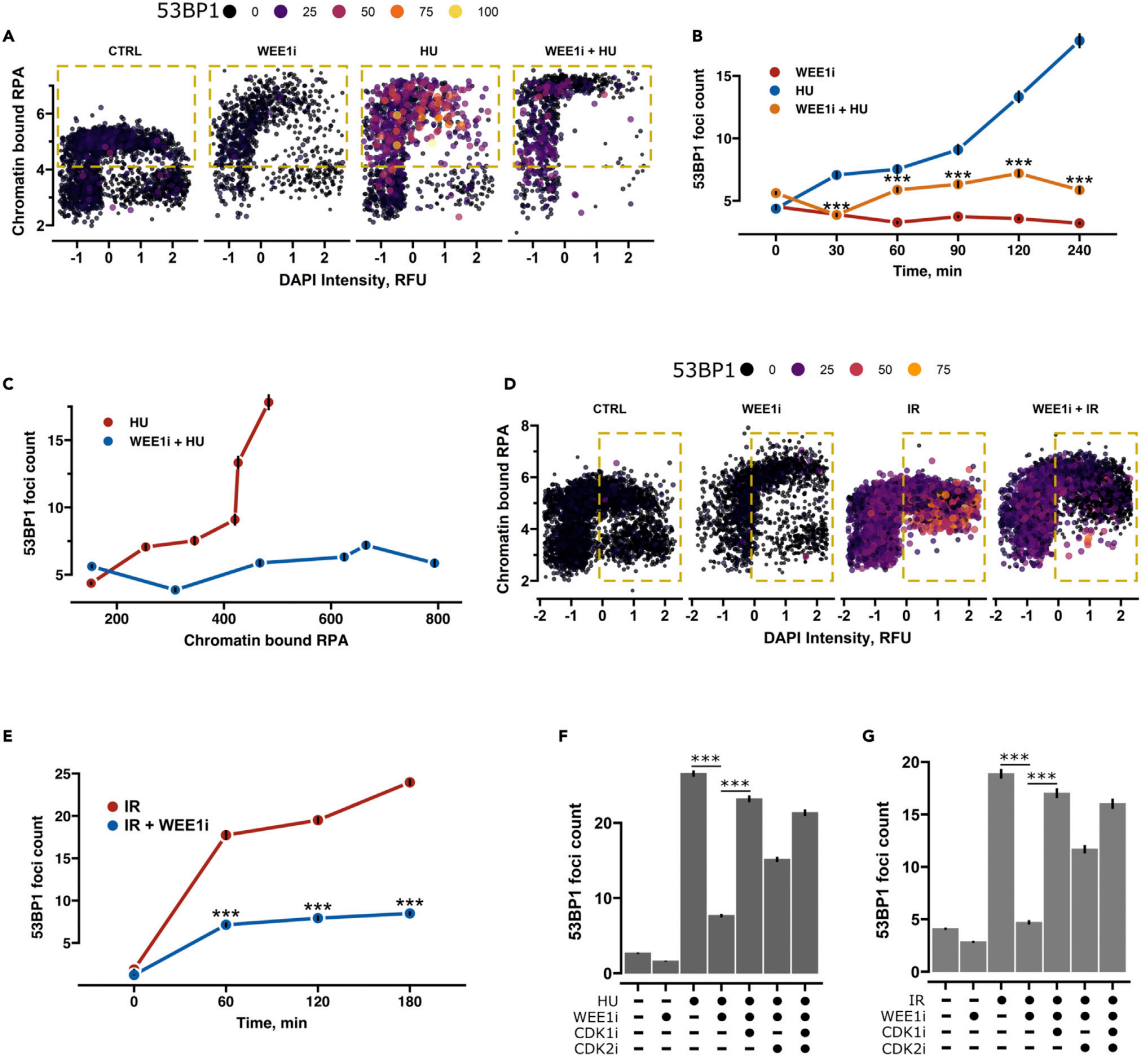
WEE1 promotes the genotoxic stress-induced recruitment of 53BP1 to chromatin by limiting CDK activity (A) Scatterplot showing cell cycle distribution based on DAPI and mean chromatin-bound RPA intensity. Color intensity represents 53BP1 foci count. Yellow dashed segment indicates the cell population used for summary statistic calculation. Cells were treated with 4 mM of hydroxyurea (HU) for 4 h, WEE1i (adavosertib) was used at 1 μM concentration. N = 2, n > 900. (B) Dot plot showing 53BP1 foci mean values resolved by time with HU, WEE1i, or HU + WEE1i treatment. N = 2, n > 900, p-values were calculated with Kolmogorov-Smirnov test. ∗∗∗ indicates p-val <0.0001. HU and HU + WEE1i conditions are compared. Black bars indicate the SE. (C) Plot showing the correlation between mean chromatin-bound RPA intensity and mean 53BP1 foci count. N = 2, n > 900, black bars indicate the SE. (D and E) As in A and B, but cells were treated with 2 Gy of IR. The 3 h post IR time point is shown in D. N = 2, n > 2500. (F and G) Bar plots showing mean 53BP1 foci values with addition of CDK1- (RO-3306 - 10 μM) and CDK2 (CDK2 inhibitor II - 5 μM)-specific inhibitors. Black bars indicate SE. N = 2, n > 500, p values were calculated with Kolmogorov-Smirnov test. ∗∗∗ indicates p-val <0.0001. All experiments were carried out at least in biological duplicate and one representative replicate is shown. N indicates the number of times the experiment has been replicated and n notes the minimal amount of cells quantified per condition.

Based on the observed phosphorylation clusters, we hypothesized that adavosertib could trigger untimely hyperphosphorylation of RIF1 and 53BP1 suppressing their function. To determine if this was indeed the case, we treated cells with genotoxic agents in combination with adavosertib to allow easy detection of 53BP1 localization. Furthermore, we utilized quantitative image-based cytometry to quantify the levels of replication stress (chromatin-bound RPA), DNA damage (γH2AX), and 53BP1 foci formation. Since RIF1 association with DNA damage sites is dependent on 53BP1,^3^^,^^6^ we focused on 53BP1 recruitment. By treating the cells with hydroxyurea (HU) for variable periods of time (Figure S5A), we observed a marked increase in chromatin-bound RPA intensity indicating replication stress and in turn elevated numbers of 53BP1 foci primarily in replicating cells (Figures 7A and S5B. As specific cell cycle populations are affected, we segmented the cells based on the cell cycle profile and excluded segments with low dynamic range prior to summary statistic calculation (Figure S5C and S5D). WEE1 inhibition alone resulted in comparable levels of chromatin-bound RPA and γH2AX as HU (Figures S5E and S5F) though no increase in 53BP1 foci was detected (Figure 7A). Importantly, the addition of adavosertib prevented the HU-induced recruitment of 53BP1 to chromatin already from the 30 min time point (Figure 7B). Furthermore, both the total 53BP1 foci intensity, as well as foci size, were decreased suggesting that observed effects were not due to delocalization of 53BP1 to a fewer number of foci (Figure S5G). Adavosertib addition did not limit HU-induced RPA accumulation indicating the presence of replication stress (Figure 7C). To test if the observed effect on 53BP1 was specific for replication stress, we treated cells with ionizing radiation (IR) and monitored 53BP1 foci levels at 1, 2, and 3 h post IR through cell cycle phases (Figure S6A). IR increased the 53BP1 foci formation throughout the cell cycle (Figures 7D, S6B, and S6C). In line with our previous observations for HU, inhibition of WEE1 prior to IR resulted in the suppression of 53BP1 foci formation. Notably, this was apparent in S and G2 phase cells where CDK1 and CDK2 are most active (Figures 7E and S5C). Moreover, the identified role is not cell line specific as adavosertib led to a reduction in 53BP1 foci formation in both transformed and non-transformed cell lines (Figures S7A–S7E).

Our data indicate that the genotoxic stress-induced association of 53BP1 with chromatin is promoted by WEE1. To determine if this is due to CDK activity control, we performed rescue experiments with inhibitors designed to target CDK1 or CDK2.^49^^,^^50^ We used inhibitors as CDK1 is essential rendering depletion or deletion unsuitable. Notably, co-treatment of adavosertib with CDK1 or CDK2 inhibitors could rescue 53BP1 foci formation in both HU- and IR-treated cells indicating that reduction of 53BP1 foci upon WEE1 inhibition was dependent on CDK activity (Figures 7F and 7G). CDK inhibitors have poor selectivity and generally affect multiple CDKs, making it impossible to confidently separate CDK2 and CDK1 function.^49^^,^^51^ The CDK1 inhibitor was more efficient in restoring 53BP1 foci compared to CDK2. This could indicate that CDK1 has a more marked role; however, the stronger effect could be due to inhibitor chemical properties as the CDK1 inhibitor exhibited a stronger effect on CDK substrate phosphorylation (Figure S7F). Albeit we cannot confidently pinpoint impacts exclusively to either CDK1 or CDK2, the observed suppression of 53BP1 foci in interphase is clearly CDK driven.

## Discussion

### Summary of findings

Our study utilized deep phosphoproteome profiling in combination with bioinformatic analysis to reveal downstream kinase-mediated signaling pathways upon the inactivation of WEE1 with adavosertib. Inhibition of WEE1 rapidly triggers high CDK1 and CDK2 activity, which in turn could affect other kinase signaling pathways. We identified the specific temporal WEE1-regulated phosphorylation patterns of CDK substrates and features that are governing them. Furthermore, we show that parallel to its mitotic role, WEE1 can indirectly control factors involved in DNA replication and repair. Specifically, we identified that dysregulation of the WEE1-dependent CDK activity impacted 53BP1 by blocking its association with chromatin.

### WEE1 and the kinome

WEE1 serves a vital function in controlling CDKs, which are pivotal kinases that are intertwined with numerous cellular functions. Loss of WEE1 activity rapidly induces high levels of CDK-driven DNA damage; for this reason, fast inactivation of WEE1 is required to confidently elucidate the role of WEE1 without confounding effects.^8^^,^^14^ This renders most genetic approaches, such as siRNA depletion or CRISPR knock-outs unfeasible, as cells start accumulating γH2AX already 4 h after WEE1 inactivation (Figure S1A). Accordingly, we used chemical inhibition of WEE1 with adavosertib, which allows us to quickly inactivate WEE1 and minimize the secondary effects,^17^ though results may be impacted by potential inhibitor off-target effects. Two previous studies have carried out *in vitro* kinome profiling for adavosertib and assessed both binding and effects on kinase activity.^26^^,^^27^ From our kinase activity inference, we observed that inhibition of WEE1 led to the suppression of CSNK2A1, MTOR, AKT, and DYRK2 kinase activities (Figure 2D). Although adavosertib could bind to CSNK2A1, it did not affect the activity of the kinase, suggesting that the decreasing CSNK2A1 activity (Figures 2D and 2E) was not due to off-target effects of the inhibitor. Overall, these newly identified functional kinase links provide a foundation for further explorations, which can shed light on how WEE1 impacts the function of non-CDK kinases.

### Prediction of potential CDK phosphorylation sites

An important aspect of our study is that the controlled dysregulation of CDK activity facilitates detection of putative new phosphosites. We constructed PSSM based on previously identified CDK1 and CDK2 phosphorylation sites and combined it with our experimental data to predict tentative CDK phosphorylation sites (Figure 4A). We identified a total of 628 potential CDK sites with high accuracy for our prediction algorithm using ROC analysis (Figure S4C). However, it is possible that over-fitting of the training data occurred, as the same motifs used for PSSM generation are used for the logistic regression model. More advanced algorithms that utilize neural networks or ensemble learning approaches could in principle be used to further increase the prediction accuracy.^52^^,^^53^ However, we chose PSSM, as they have recently been successfully used to predict kinase substrates.^32^^,^^54^^,^^55^ Furthermore, PSSM also displays marked simplicity and a high degree of interpretability, which is not present in the before-mentioned algorithms. We affirmed the prediction by cross-referencing our tentative CDK sites with an independent study.^34^ A majority of our new sites were indeed downregulated after CDK1 inhibition (Figures 4D and 4E). The identified sites are positioned on proteins involved in fundamental cellular pathways, and this will aid researchers studying not only CDK activity but also fundamental cellular processes.

### Identification of CDK substrate population with distinct phosphorylation dynamics

The timing of CDK-driven substrate phosphorylation plays a paramount role in the ordering of the cell cycle. Ectopic target phosphorylation has been shown to perturb the order of cell cycle stages,^40^^,^^56^ underlining the importance of accurate phosphorylation timing. In yeast, cell cycle-specific CDK substrate phosphorylation is in part achieved by exploiting cyclin-binding motifs present on early (G1/S) cell cycle phase targets.^40^ Accordingly, we observed two distinct phosphorylation dynamic profiles (Figures 5A and 5B); however, we did not observe significant differences in the availability of potential Cy motifs at functionally relevant distances from the phosphorylation site (Figure 5E). As Cy motif functional validation is limited, we used a regular expression to identify potentially Cy motifs. This approach identifies all instances in linear amino acid sequences that match a present Cy motif pattern [R-x-L] though many of the matched sequences might be not functional, as they do not account for the 3D protein structure. However, we noted a significant difference in amino acid frequencies flanking the phosphorylation sites (Figures 5C and 5D). Specifically, we observed a preference for serine over threonine residues and increased lysine frequency in position +3 from the phosphorylation site. Relative to CDK1, CDK2 has a higher preference for the lysine residue,^37^ suggesting that the fast phosphorylation dynamics could largely be mediated by CDK2 (Figures 5C and 5D).

### Inactivation of WEE1 triggers rapid phosphorylation of DNA replication and repair factors

Mechanisms that maintain genome integrity not only play a role in preventing malignant transformation but they are also targeted in cancer therapy.^1^ CDK activities are known to play dual roles in DNA repair by both promoting and suppressing the function of DNA repair proteins.^57^^,^^58^^,^^59^^,^^60^ We discovered that adavosertib triggers hyperphosphorylation of residues in the C-terminal region of 53BP1 and RIF1 (Figures 6C and 6D), which have been linked with inhibitory roles. In addition to direct phosphorylation by CDK1, the inactivation of 53BP1 and RIF1 is promoted by PLK1 and AURKB, respectively. However, in both cases, this is dependent on CDK activity.^45^^,^^46^^,^^47^^,^^48^^,^^61^^,^^62^ In particular, 53BP1 C-terminal hyperphosphorylation takes place within a region necessary for 53BP1 localization to the damaged chromatin (also termed a focus forming region, 1220-1711aa). CDK-dependent hyperphosphorylation of this region has been shown to prevent 53BP1 localization to the sites of DNA damage and thus inhibit DNA repair. In physiological conditions, this occurs exclusively during mitosis in order to prevent toxic sister telomere fusions.^46^ Notably, here we described that the inhibition of WEE1 suppressed the recruitment of 53BP1 to chromatin after DNA damage by analogous mechanism during the interphase (Figures 7B and 7E). Accordingly, WEE1 inhibition has been shown to hinder DNA repair;^58^^,^^60^ thus, enhanced 53BP1 phosphorylation can potentially sensitize cells to genotoxic agents that induce DSBs or cause replication stress.^43^^,^^63^^,^^64^ More recently, it was reported that both 53BP1 and RIF1 can protect stalled DNA replication forks from excessive DNA2 exonuclease-mediated degradation.^43^^,^^63^ Accordingly, we have previously shown that WEE1 guards against the degradation of nascent DNA at stalled replication forks by DNA2.^15^ Thus, WEE1 may promote the protection of stalled forks from DNA2-driven degradation by specifically limiting CDK-driven phosphorylation of 53BP1 and RIF1. Considering the marked impact on 53BP1 recruitment, adavosertib triggers high CDK activity which not only causes DNA damage but also hinders mechanisms that should mitigate it.

### WEE1 inhibition and cancer therapy

Although WEE1 has a major role in regulating the G2/M checkpoint, its role in S phase is also important.^65^ Evidently, adavosertib can synergize with other cancer therapy agents that induce replication stress such as gemcitabine, CHK1, ATR, or PARP1 inhibitors, potentially by exacerbating replication stress to intolerable levels.^18^^,^^19^^,^^20^^,^^66^ Accordingly, WEE1 inhibition has shown promising results in ovarian and pancreatic cancers, which have high basal levels of replication stress.^21^^,^^22^ Past studies have addressed the role of WEE1 in S phase showing that adavosertib triggers increased origin firing and nucleotide depletion by affecting MCM4, RIF1, and RRM2^8^^,^^13^^,^^48^ and chromosome fragmentation by regulating SLX4/MUS81.^14^ However, its function in S phase is not completely understood. Here, we supply a resource of WEE1-dependent phosphorylation sites on key DNA replication and repair proteins. Furthermore, we identified numerous phosphorylation sites in cellular pathways such as mitosis and mRNA metabolism, which are not explored in this study. Overall, our findings indicate yet undescribed roles of WEE1-controlled CDK activities with potentially substantial impact on the clinical application of WEE1 inhibitors.

### Limitations of the study

In this study, we utilize computational analysis of large-scale phosphoproteomic datasets. By utilizing different algorithms, we establish links between multiple kinases and the WEE1-CDK axis and predict putative CDK sites. Certain limitations of our chosen data acquisition and analysis approach have to be highlighted. In accordance with previous studies, we use a relatively high concentration of adavosertib, reminiscent of the blood serum concentration in clinical studies.^8^^,^^15^^,^^23^^,^^27^^,^^28^^,^^34^ Albeit such concentrations have been they routinely used, they increase the risk of convoluting the data with off-target effects. Here, PSSM-based filtering plays a pivotal role as it allowed us to rule out sites that are unlikely CDK substrates based on the surrounding amino acid sequence. In our validation experiments, we use CDK inhibitors, which are known to affect multiple members of CDK family and similarly to adavosertib in practice are used in high concentrations.^15^^,^^34^^,^^48^^,^^49^^,^^51^^,^^51^^,^^67^^,^^68^ Although such high concentrations may seem excessive, they are required to suppress target kinase activities efficiently;^49^ however, off-target impacts should be considered when analyzing and exploiting the findings in follow-up research. Finally, we did not consider phosphatase activities in our bioinformatic analysis, which potentially also play a role in the observed changes. Nonetheless, we anticipate that our findings will be a valuable resource and serve as basis for hypothesis generation in future studies.

## STAR★Methods

### Key resources table

**Table:** 

REAGENT or RESOURCE	SOURCE	IDENTIFIER
**Antibodies**		

γH2AX	Merck	Cat# PC130; RRID:AB_2238184
53BP1	Novus Biologicals	Cat# NB100-304, RRID:AB_10003037
BRCA1	Santa Cruz	Cat# sc-6954, RRID:AB_626761
RPA	Millipore	Cat# MABE285, RRID:AB_11205561
CDK substrate	Cell Signaling Technology	Cat# 9477, RRID:AB_2714143
CDK pY15	Cell Signaling Technology	Cat# 9111, RRID:AB_331460
CDK1	Cell Signaling Technology	Cat# 77055, RRID:AB_2716331
Actin	Millipore	Cat# MAB1501, RRID:AB_2223041
HRP anti-mouse	Vector Laboratories	Cat# PI-2000, RRID:AB_2336177
HRP anti-rabbit	Vector Laboratories	Cat# PI-1000, RRID:AB_2336198

**Chemicals, peptides, and recombinant proteins**		

RO-3306	CalBioChem	Cat# 217699; CAS:872,573-93-8
CDK2 inhibitor II	Santa Cruz	Cat# sc-221409; CAS:222,035-13-4
Adavosertib/MK-1775/AZD1775	SelleckChem	Cat# S1525; CAS:955,365-80-7
Hydroxyurea	Merck	Cat# H8627; CAS:127-07-1
TMTpro™ 16plex Label Reagent Set	Thermo Fisher	Cat# A44520

**Critical commercial assays**		

MagReSyn® Ti-IMAC HP	LabLifeNordic	Cat# MR-THP002

**Deposited data**		

Proteomics data	This paper	MassIVE: PXD036374; MassIVE: PXD036373
Custom code used for proteomics data	This paper	Zenodo: https://doi.org/10.5281/zenodo.6344325
QIBC data and analysis code	This paper	Zenodo: https://doi.org/10.5281/zenodo.7022473

**Experimental models: Cell lines**		

U2OS	ATCC	HTB-96
MFC10A	ATCC	CRL-10317
RPE	ATCC	Laboratory of Krister Wennerberg
OVCAR3	ATCC	Laboratory of Krister Wennerberg
OVCAR8	ATCC	Laboratory of Krister Wennerberg

**Software and algorithms**		

Visual Studio Code	Microsoft	https://visualstudio.microsoft.com
R project	N/A	https://www.r-project.org
Spotfire 11	Tibco	RRID:SCR_008858
ProteomeDiscoverer	Thermo Fisher	RRID:SCR_014477
Tidyverse	(Wickham et al., 2019)^75^	https://tidyverse.tidyverse.org/
ggprism	(Charlotte Dawson 2021)	https://doi.org/10.5281/zenodo.4556067
ggplot2	(Wickham et al., 2016)	https://ggplot2.tidyverse.org
ClusterProfiler	(Wu et al., 2021)^74^	https://doi.org/10.1089/omi.2011.0118
VennDiagram	(Chen and Boutros, 2011)^76^	https://cran.r-project.org/web/packages/VennDiagram/index.html
OmnipathR	(Türei et al., 2016)^25^	https://omnipathdb.org/
Mfuzz	(Kumar and Futschik, 2007)^70^	http://mfuzz.sysbiolab.eu/
ggseqlogo	(Wagih, 2017)^71^	https://omarwagih.github.io/ggseqlogo/

### Resource availability

#### Lead contact

Further information and requests for resources and reagents should be directed to and will be fulfilled by the lead contact, Claus Storgaard Sørensen (claus.storgaard@bric.ku.dk).

#### Materials availability

This study did not generate new unique reagents.

### Experimental model and subject details

#### Cell lines

Human osteosarcoma cell line U2OS and retinal pigment epithelial RPE cell line were cultured in Dulbecco’s Modified Eagle’s medium (DMEM; Gibco) supplemented with 10% foetal bovine serum (FBS; Cytiva) and 1% Penicillin-Streptomycin (10,000 U/mL; Gibco). Human mammary gland cell line MCF10A was cultured in Dulbecco’s Modified Eagle Medium/Nutrient Mixture F-12 (DMEMF12; Gibco) supplemented with 5% horse serum (Gibco), 1% Penicillin-Streptomycin, 20 ng/mL EGF (PeproTech), 10 mg/mL insulin (Sigma-Aldrich), 0.5 mg/mL hydrocortisone (Sigma-Aldrich), and 100 ng/mL cholera toxin (Sigma-Aldrich). High grade serous ovarian cancer cell lines OVACAR3 and OVCAR8 were cultured in Roswell Park Memorial Institute 1640 medium (RPMI1-1640, Gibco) supplemented with 10 % feotal bovine serum and 1 % Penicillin-Streptomycin. All cells were cultured in 37°C with 5% CO2 and checked for mycoplasma infection regularly.

### Method details

#### MS sample preparation

U2OS cells were treated for 0, 20, 40, 60 and 90 min (n=3) for the time-course experiment and for 0 or 90 min (n = 4) for the single time point dataset with 1 μM of adavosertib. After treatment, the cells were subsequently harvested by scrapping and processed according to (Kulak et al., 2014). Briefly, harvested cells were lysed using 100 μL of lysis buffer (6 M Guanidinium Hydrochloride, 10 mM TCEP, 40 mM CAA, 50 mM HEPES pH8.5). Samples were boiled at 95°C for 5 min, after which they were sonicated on high for 5x 30 seconds in a Bioruptor sonication water bath (Diagenode) at 4^o^C. After determining protein concentration with Pierce™ Rapid Gold BCA Protein Assay Kit (Thermo Fisher), 200ug were taken forward for digestion. Samples were diluted 1:3 with 10% Acetonitrile, 50 mM HEPES pH 8.5, LysC (MS grade, Wako) was added in a 1:50 (enzyme to protein) ratio, and samples were incubated at 37^o^C for 4hrs. Samples were further diluted to 1:10 with 10% Acetonitrile, 50 mM HEPES pH 8.5, trypsin (MS grade, Promega) was added in a 1:100 (enzyme to protein) ratio and samples were incubated overnight at 37^o^C. Enzyme activity was quenched by adding 2% trifluoroacetic acid (TFA) to a final concentration of 1%. Prior to TMT labeling, the peptides were desalted with the use of SOLAμ™ SPE Plate (Thermo Fisher). The sorbent was activated by 40μl of 100% Methanol (HPLC grade, Sigma), then 40μ of 80% Acetonitrile, 0.1% formic acid. The plate was subsequently equilibrated 2x with 40 μL of 1%TFA, 3% Acetonitrile, after which the samples were loaded. The plates were then washed 2 times with 200 μL of 0.1% formic acid and the bound peptides were eluted into clean 500 μL Eppendorf tubes using 40% Acetonitrile, 0.1% formic acid. The eluted peptides were concentrated in an Eppendorf Speedvac and re-constituted in 50 mM HEPES (pH8.5) for TMT labeling. TMTPro 16plex (Thermo Fisher) was used according to manufacturer’s instructions. After labeling the peptides pooled with the same ratio and 200 μL of Ti-IMAC HP (ReSyn Bioscience) was used for enrichment of phosphopeptides. The Ti-IMAC particles were equilibrated with 200 L of 70% ethanol for 5 min and subsequently with 200 μL 1% NaNH_3_ solution for 10 min. Particles were then washed three times with 200 μL of 80% Acetonitrile, 1M glycolic acid, 5% TFA . Pooled peptides that have been diluted 1:1 with 80% Acetonitrile, 1M glycolic acid, 5% TFA were loaded onto the particles and incubated for 30 min with gentle agitation. Supernatant was then removed and kept as the proteome sample. The particles were washed twice with 400μL of 80% Acetonitrile, 1% TFA for 2 min and twice 400 μL of 10% Acetonitrile 0.1% TFA. The phosphopeptides were then eluted in three rounds by adding 100 μL of 1 % NaNH_3_ solution for 20 min. The eluted phosphopeptide solution was then acidified with 40 μL of TFA and the supernantant proteome samples were diluted to reduce the Acetonitrile concentration to 5%. Peptide solutions were then desalted with the use of SOLAμ™ SPE Plate and subsequently peptides were concentrated in an Eppendorf Speedvac. Concentrated peptides were stored in -80^o^C.

Prior to mass spectrometry analysis, the peptides were fractionated using an offline ThermoFisher Ultimate3000 liquid chromatography system using high pH fractionation (5mM Ammonium Bicarbonate, pH 10) at 5μl/min flowrate. 30μg of peptides were separated over a 120 min gradient (5% to 35% Acetonitrile), while collecting fractions every 120 sec. The resulting 60 fractions were pooled into 30 final fractions, acidified to pH < 2 with 1% TFA and loaded onto EvoSep stagetips according to manufacturer’s protocol. For the enriched phospho-peptides, the number of fractions was adjusted to the total amount of phospho-peptide available, to ensure the collection of ∼500ng per fraction.

#### MS data acquisition

##### TMT-labeled samples

For each fraction, peptides were analysed using the pre-set ’30 samples per day’ or ’15 samples per day’ method on the EvoSep One instrument. Global proteome peptides were eluted over a 44-min gradient, and analysed on a Q-Exactive Exploris 480 instrument (Thermo Fisher Scientific) running in a DD-MS2 method. Full MS spectra were collected at a resolution of 120,000, with an AGC target of 3×106 or maximum injection time of 50 ms and a scan range of 350–1500 m/z. The MS2 spectra were obtained at a resolution of 45,000, with an AGC target value of 1×105 or maximum injection time of 86 ms, a normalized collision energy of 32 and an intensity threshold of 1e5. First mass was set to 110 m/z to ensure capture of the TMT reporter ions. Dynamic exclusion was set to 60 s, and ions with a charge state <2, >6 and unknown were excluded. For phosphorylated peptides, the gradient length was doubled to 88mins. MS performance was verified for consistency by running complex cell lysate quality control standards, and chromatography was monitored to check for reproducibility.

#### TMT quantitative proteomics analysis

The raw files were analyzed using Proteome Discoverer 2.4. TMT reporter ion quantitation was enabled in the processing and consensus steps, and spectra were matched against the 9606 Human database obtained from UniProt. Dynamic modifications were set as Oxidation (M), Deamidation (N,Q) and Acetyl on protein N-termini. Cysteine carbamidomethyl (on C residues) and TMTPro (on peptide N-termini and K residues) were set as static modifications. All results were filtered to a 1% FDR, and protein quantitation done using the built-in Reporter Ions Quantifier with statistical significance testing was done with the built-in t-test.

#### Label-free quantification

Samples for label-free quantification were process as described above. Both phosphoproteome and proteome peptides were analyzed with a “20 samples per day” method on the EvoSep One instrument and analysed on Q-Exactive Exploris 480 instrument (Thermo Fisher Scientific) running a high-resolution MS1 data-independent acquisition method. Full MS spectra were collected at 120 000 with AGC target of 3x10ˆ6 or maximum injection time of 50 ms. Scan range from 400-1000 m/z was used. The MS2 spectra were obtained with 60,000 resolution, with a AGC target of 10e5. 75 windows windows of 8 m/z were used with a MS1 scan every 200 m/z. The raw files were the analysis with Spectronaut 16 software.

#### Data normalization and statistical testing

Protein group abundances were used for the proteome and phosphopeptide abundances for the phosphoproteome. Data normalization was carried out in two steps. First, sample loading was adjusted by multiplying the abundances by a sample specific scaling factor:

(Equation 1) $${SF}_{i}=\frac{\bar{AS}}{{AS}_{i}}$$

Where ${SF}_{i}$ is the scaling factor for sample *I,* $\bar{AS}$ is the mean abundance sum from all the samples and ${AS}_{i}$ is the abundance for sample *i.* Subsequently, artificial internal reference standards were generated protein/peptide specific scaling factor for each replicate and used for normalization to reduce the variation due to biological replicate processing:

(Equation 2) $${ARS}_{i,j}=\frac{\sum^{n}A_{i}}{n}$$

(Equation 3) $$\bar{{ARS}_{j}}=\frac{\sum^{m}{ARS}_{j}}{m}$$

(Equation 4) $${ARSSF}_{i,j}=\frac{\bar{{ARS}_{j}}}{{ARS}_{i,j}}$$

(Equation 5) $$A_{i,j}^{ARS}=A_{i,j}\cdot {ARSSF}_{i,j}$$

Where ${ARS}_{i,j}$ is the artificial reference standard for protein/peptide *j* in biological replicate *i* and *n* is the number of unique conditions (time points) in the biological replicate. Biological replicate scaling factors are then calculated similar as for sample loading with Equations 3 and 4. Where $\bar{{ARS}_{j}}$ is the artificial reference standard for the whole dataset, where *m* is the number of biological replicates and ${ARSSF}_{i,j}$ is the scalling factor protein/peptide *j* in biological replicate replicate *i.* Finally, protein/peptide abundances are corrected $A_{i,j}^{ARS}$ by multiplying by the scaling factor according to Equation 5. Significant altered phosphopeptides were identified with the use of the limma statistical package.^69^

#### Kinase activity inference

Relative kinase activity was calculated by first annotating the detected phosphorylation with kinases based on the PhosphoSitePlus and SIGNOR databases downloaded via the Omnipath.^25^ Phosphosite abundances were scaled peptide-wise to prevent overall site abundance from inferring with the inference. Relative kinase activity was calculated by combining the values with the Stouffer *Z* score method for each sample:

(Equation 6) $${RKA}_{k}=\frac{\sum_{i=1}^{n}S_{n}}{\sqrt{n}}$$

Where, *RKA* is the relative kinase *k* activity, *S* is the scaled abundance of a phosphopeptide for a specific kinase *k* and *n* is the number of phosphosites that correspond to kinase *k* in the dataset. A mean RKA score was calculated for each sample with a linear model of the form y=β·S for each time point, under the assumption that the RKA scores follow a normal distribution. Where y a vector containing the RKA scores for a specific kinase ,S the design matrix indicating, which score belongs to which sample, and β a vector containing the fitted mean RKA scores for each condition.

#### Protein phosphorylation dynamic analysis

To identify patterns of phosphorylation dynamic fuzzy c-means clustering was used.^70^ Before clustering abundances were scaled peptide-wise. The optimal parameters were calculated with the built in functions in the package. The clustering was carried out with parameters n = 8 and c = 2. Michaelis-Menten equation was the used to model the clusters 7 and 8.

(Equation 7) $$y=\frac{V_{m}\cdot x}{K+x}$$

y – is the scaled phosphopeptide intensity and x - time after WEE1 inhibition. Non-linear least squares were used to fit the optimal parameters for *V*_*m*_ and *K.*

#### Binomial probability model for motif analysis

Binomial probability model^31^ was used to generate the position specific scoring matrixes (PSSMs) and the “ggseqlogo” package was used to visualize the sequence motifs.^71^ The residue score at a certain position is assessed by calculating the log odds of the ratio between probability of over or under-represenation:

(Equation 8) $${RS}_{a}\left(K_{a},N,p_{a}\right)=-\log_{10}\frac{P_{a}\left(k,\forall k\geq K_{a}|N,p_{a}\right)}{P_{a}\left(k,\forall k\leq K_{a}|N,p_{a}\right)}$$

RS is the amino acid *a* residue score a specific position in the motif, *N* – is the total number of all residues at that position (or the total numbers of amino acids sequences used), *K* – is the observed count of residue *a* at a specific position, *p* – is the background amino acid frequency of residue *a*. The respective probabilities are calculated as follows:

(Equation 9) $$P_{a}\left(k,\forall k\geq K_{a}|N,p_{a}\right)=\sum_{k=K_{a}}^{N}Bin\left(k,N,p_{a}\right)$$

(Equation 10) $$P_{a}\left(k,\forall k\leq K_{a}|N,p_{a}\right)=\sum_{k=0}^{N}Bin\left(k,N,p_{a}\right)=1-\sum_{k=K_{a}}^{N}Bin\left(k,N,p_{a}\right)$$

#### Background amino acid frequency calculation

Phosphorylation sites in disordered protein regions, to avoid enriching our motifs with residues whose frequencies are highly dependent on protein disorder, we calculated the disordered scores for all protein amino acid sequences in the human proteome obtained from UniProt,^72^ by using IUPred2A algorithm via the “idpr” R package (McFadden and Yanowitz, not reviewed). Only residues that had disorder score higher than 0.4 were used to calculate the background amino acid frequency as follows:

(Equation 11) $$F_{i}=\frac{A_{i}}{T}$$

*A*_*i*_ is the count of an amino acid *i*, T is the count of total amino acids. Both values are after disorder propensity filtering.

#### Position specific scoring matrix construction

We predicted kinases for the unknown phosphorylation sites by constructing position specific scoring matrixes (PSSMs) for the amino acid sequences flanking the known phosphorylation sites. We extracted the phosphorylation sites from the PhosphositePlust and SIGNOR databases via the Omnipath. Sites with only a curation effort of above 2 were considered and PSSM were generated only for kinases that had at least 20 phosphorylation sites after the filtration. Amino acid sequences surrounding the phosphorylation site (+/- 10) were then extracted from the full-length protein amino acid sequences that have been downloaded from the UniProt database. The binomial probability model was then used to calculate scores for every amino acid for all the possible positions generating a 21x20 matrix. The phosphorylation sites were then scored for each kinase with the following equation:

(Equation 12) S=∑RS(a,b)

Where *S* is the score of a phosphorylation site for a given kinase PSSM and *RS* is binomial probability model score for amino acid *a* at position *b.* To ensure comparability between kinases logistic regression is used to calibrate the kinase scores. The known phosphorylation sites (without curation effort cut-off) were then separated into 80% training and 20 % testing dataset. Since, some of the testing dataset examples were used to generate the PSSM, the accuracy characteristics will be favorably skewed, however the test set serves a good sanity check for the logistic regression step. Since logistic regression is sensitive to imbalanced datasets, we use down sampling, to match the number of positive and negative instances that are used for logistic regression. Generalized linear modeling was used to fit the logistic regression function with a logit link function:

(Equation 13) $$P\left(S\right)=\frac{e^{\beta_{0}+\beta_{1}S}}{1+e^{\beta_{0}+\beta_{1}S}}$$

(Equation 14) $$logit\left(S\right)=\beta_{0}+\beta_{1}S$$

CDK1, CDK2 and CDK5 prediction accuracy was the analysis by receiver operating characteristic analysis with the used of the “pROC” package.^73^

#### Comparison of CDK substrate phosphorylation dynamics

To identify preferences in the amino acid residues flanking the phosphorylation site amino acid frequencies between the “slow” and “fast” CDK substrates directly compared. To obtain an uncertainty measure in our comparisons we utilized bootstrapping. Phosphorylation sites with their flanking amino acid sequences were samples from the clusters 100 times, to generate resampled datasets with different compositions. Then amino acid frequency was calculated for each dataset and the “slow” cluster frequencies were subtracted from the “fast”. The mean and standard deviation of the frequency differences was then calculated for each amino acid in all possible positions. Under the null hypothesis the frequency difference should be 0, to test this we calculated the probability of observing the calculated frequency difference from a normal distribution parametrized by a mean of 0 and an amino acid position specific standard deviation.

The regular expression – [R/K]-x-[L/V/I] was used to identify all the potential Cy motifs in the full-length protein amino acid sequence. The closes Cy motif to the detected phosphorylation site was extracted on both left and right sides of the modification. The distances were visualized by binning them into a histogram or generating a cumulative mass function. Statistical significance was tested with the Kolmogorov-Smirnov test.

#### Gene-set over-presentation analysis

Gene-set overrepresentation analysis was carried out with the use of the ClusterProfiler R package.^74^ The detected phosphoproteins were used as the background for the representation analysis. Biological pathways (BP) and cellular compartmentalization (CC) gene-sets tested for enrichment. As these gene-set do not account for PTMs, simply protein accession entries were used in place of the modification.

#### Quantitative image-based cytometry

Cells growing on 96-well microplates (Greiner-BIO) were treated with different combinations of drugs and genotoxic agents for variable time intervals. After the appropriate treatment, the media was quickly removed and the cells were incubated in pre-extraction buffer (25 mM HEPES, pH 7.5, 50 mM NaCl, 1 mM EDTA, 3 mM MgCl2, 300 mM sucrose, and 0.5% Triton X-100) on ice for 2 min and immediately fixed in formaldehyde 4% (VWR) for 10 min at the room temperature. For the analysis of the mitotic cell number the pre-extraction step was omitted. Primary antibodies (γH2AX 1:300; Cell Signaling Technology, RPA 1:200; Sigma-Aldrich and 53BP1 1:1000, Novus Biotechnology) were diluted in filtered DMEM containing 10% FBS and 5% Bovine Serum Albumin (BSA; Sigma). Incubations with the primary antibodies were performed at RT for 1 h. Microplates were washed three times with 0.05% PBS-Tween20 and incubated in DMEM/FBS/BSA containing secondary fluorescently labeled antibodies (Alexa Fluor dyes (1:1000; Thermo Fisher Scientific) and DAPI (0.5 mg/mL; Sigma-Aldrich) for 1 h at RT. Images were obtained automatically with the ScanR acquisition software controlling a motorized Olympus IX-83 wide-field microscope. Olympus Universal Plan Super Apo 10x Objective was used for all QIBC data. However, 53BP1 foci images were obtained with a 40x objective. Images were processed and quantified using the ScanR image analysis software for total nuclear pixel intensities for DAPI (Arbitrary units: A.U.) and mean (total pixel intensities divided by nuclear area) nuclear intensities (A.U.) for γH2AX, chromatin-bound RPA and 53BP1 foci. Further analysis was then carried out with R statistical software. Kolmogorov-Smirnov test was used to establish statistical significance by comparing the signal intensity distributions.

#### Immunoblotting

Cells were lysed in RIPA (Sigma) buffer containing EDTA free protease inhibitor cocktail (Roche) and 2mM DTT. Lysates were sonicated with Bioruptor sonication device for 10 cycles 30 s on and 30 s off intervals. Lysates were centrifuged for 15 min at 20 000 RFC at 4C. Protein concentration was then measured with Bradford assay and adjusted accordingly to ensure equal loading. Lysates were mixed with 4x LSB and boiled for 10 min at 95°C. Samples were run on NuPAGE Bis-Tris 4-12 % gels according to manufacturer instructions. Proteins were then transferred to a nitrocellulose membrane and blocked with PBS + 0.1% Tween + 5 % Milk powder + 0.01 % NaN_3_ and incubated overnight with primary antibodies at 4^o^C. The membrane was then washed 3 x 6 min in PBS + 0.1% Tween and incubated with secondary HRP conjugated antibodies for 1h at room temperature. Membranes were again washed 3x6 min with PBS + 0.1% Tween and incubated with Classico/Crescendo Western HRP substrate for 3 min. Protein bands were visualized on Amersham Hyperfilm/AGFA Curix Ortho films.

#### Data analysis

Analysis of data was carried out in the statistical R software with use of tidyverse environment^75^ unless otherwise stated. All the custom code used for the data analysis has been deposited to a repository.

### Quantification and statistical analysis

All statistical analysis was carried out in R (version 4.2.2) in the Visual Studio Code editor environment (version 1.73), all packaged used can be found in the key resources table. The statistical “limma” package was used to identify significantly altered phosphopeptides for both TMT and LFQ datasets.^69^ The number of replicates and condition information can be found in the legend of Figure 1. Phosphopeptides were considered altered if the FDR (Benjamini Hochberg's corrected) was below 5%. Relative kinase activity was calculated as described in the method section. The statistical significance threshold of 1% was calculated by assuming that the RKA scores follow a normal distribution. The precise value was generated with the used of the qnorm() function. Fuzzy c-means clustering in figure R was carried out with the “Mfuzz” package and the phosphosite sequence logos generation is described in detail in the method section (see binomial probability model for motif analysis). Prediction of CDK sites is explained in the results section and detailed explanation of the underlining algorithms can be found in the method section. The resampling approach used to compare amino acid frequency in CDK phosphosite motifs and the statistical test involved are described in the method section (see comparison of CDK substrate phosphorylation dynamics). For gene-set overrepresentation analysis (ORA) the hypergeometric test was used that is buil into the “ClusterProfiler” package.^74^ Benjamini-Hochberg (BH) was used to adjust p values for multiple hypothesis testing. To assess statistically significant differences between the QIBC data Kolmogorov–Smirnov test was used with ks.test() function in the time course experiments and t.test was used in the case where multiple cell lines were compared with the t.test() function. The notation of the results of the staticial test is given in the figure legends, along with the minimum number of cells measured in a condition and the number of times the experiment was carried out, noted by n and N, respectively.

## Data Availability

• The mass spectrometry data have been deposited to the ProteomeXchange Consortium (http://proteomecentral.proteomexchange.org) via the MassIVE partner repository with the dataset identifier PXD036373 and PXD036374. • All code used to analyze the proteomics data has been deposited (https://doi.org/10.5281/zenodo.6344325) and is publicly available as of the date of publication. • Any additional information required to reanalyze the data reported in this paper is available from the lead contact upon request.

## Supplemental information

Document S1. Figures S1–S7

Table S1. Phosphoproteomic data of the timecourse experiment, related to Figure 1

Table S2. Phophoproteomic data of the single timepoint experiment, related to Figure 1

Table S3. Combined phosphoproteomic data from both datasets, related to Figures 1 and 6

Table S4. Table of infered of relative kinase activity and correlation matrix, related to Figure 2

Table S5. Table of empirically predicted CDK sites, related to Figure 4

Table S5. Table of fold-changes of predicted CDK sites after WEE1 and CDK1 inhibition, related to Figure S4
