# Supplementary material for: Temporal phosphoproteomics reveals WEE1-dependent control of 53BP1 pathway
Source: iScience. 2022 Dec 13;26(1):105806. doi: 10.1016/j.isci.2022.105806 (PMC9827073; doi:10.1016/j.isci.2022.105806)
Supplement: Document S1. Figures S1–S7 [file mmc1.pdf]

iScience, Volume *26*

Supplemental information

Temporal phosphoproteomics reveals

WEE1-dependent control of 53BP1 pathway

Valdemaras Petrosius, Jan Benada, Olaf Nielsen, Erwin M. Schoof, and Claus Storgaard Sørensen

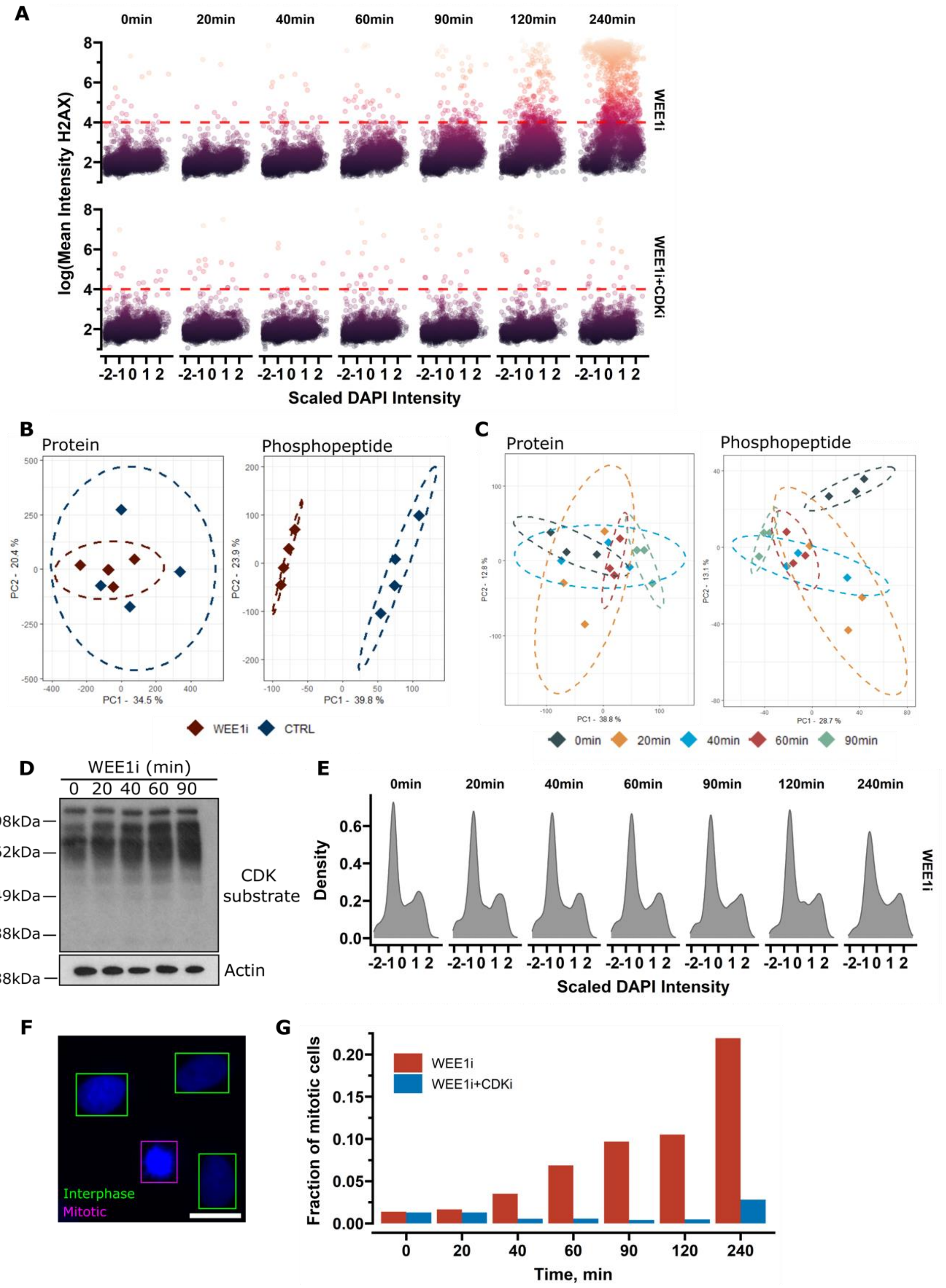
A
0min 20min 40min 60min 90min 120min 240min
log(Mean Intensity H2AX)
WEE1i
WEE1i+CDKi
Scaled DAPI Intensity
B
Protein
Phosphopeptide
PC1 - 34.5 %
PC2 - 20.4 %
PC1 - 39.8 %
PC2 - 23.9 %
WEE1i CTRL
C
Protein
Phosphopeptide
0min 20min 40min 60min 90min
D
WEE1i (min)
0 20 40 60 90
98kDa
62kDa
49kDa
38kDa
CDK substrate
Actin
E
Density
Scaled DAPI Intensity
F
Interphase
Mitotic
G
Fraction of mitotic cells
WEE1i
WEE1i+CDKi
Time, min

Figure S1. Time-resolved dynamics of cellular responses to WEE1 inhibition. Related to Figure 1. (A) Scatter plot showing γH2AX levels at different timepoints after WEE1 inhibition. CDK inhibitors are used (CDKi) to show that the observed increase in γH2AX is CDK activity dependent. Scaled DAPI intensity is plotted on the x-axis and the log transformed γH2AX intensity is on the y-axis. The plots are faceted for different timepoints and treatments. The red dashed line indicated a manually set threshold value above which a cell would be considered damaged. N=1, n>1000. **(B)** Principal component analysis plots of protein and phosphopeptide abundances for the time course experiment. The points are colored by the respective adavosertib treatment duration. Ellipses represent the confidence interval of the clustering. **(C)** Identical to **A**, but for the single timepoint dataset. **(D)** Immunoblot stained with antibodies against total CDK substrate and actin. **(E)** Scaled total DAPI density distribution at different timepoints after adavosertib addition. **(F)** Representative image from QIBC analysis showing the gating of interphase (green) and mitotic-like (red square) cells, based on their morphological features. White scale bar denotes 20 μm **(G)** Bar plot showing the cell population fraction of mitotic-like cells at different timepoints after WEE1i inhibition without or with CDK1 inhibition. QIBC and FACS experiments were carried out only once. N=1, n>1000. N indicates the number of times the experiment has been replicated and n notes the minimal amount of cells quantified per condition.

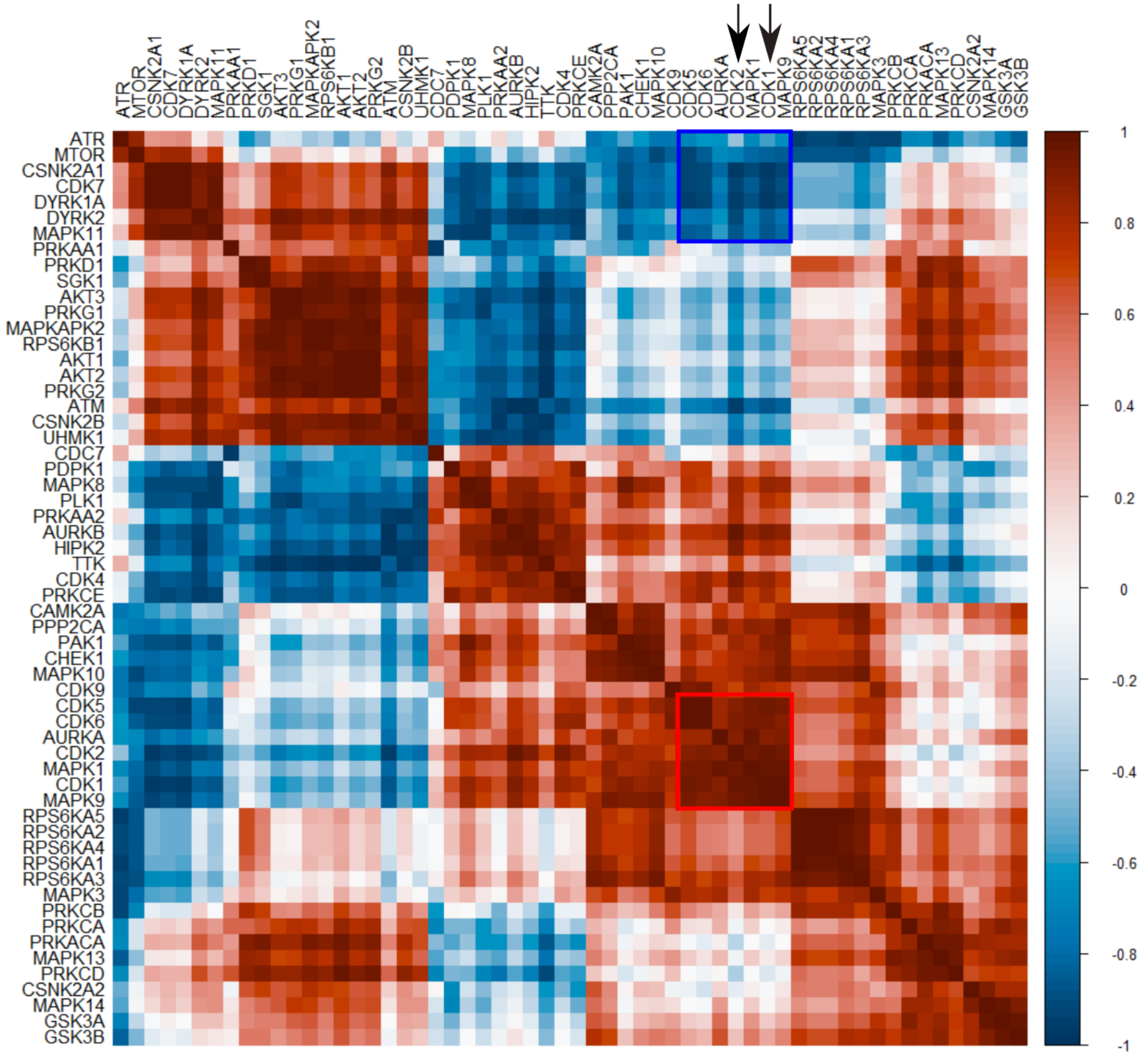

Figure S2. Kinase activity correlation map following WEE1 inhibition. Related to Figure 2 Relative kinase activity was calculated for each time point for kinases with at least 5 detected substrates. Spearman correlation was then calculated for all possible combinations. Kinases were then clustered with hierarchical clustering to identify the most correlated trends. Color indicates the correlation coefficient. Arrows denote CDK1 and CDK2. Red and blue squares indicated areas of interest in the correlation map.

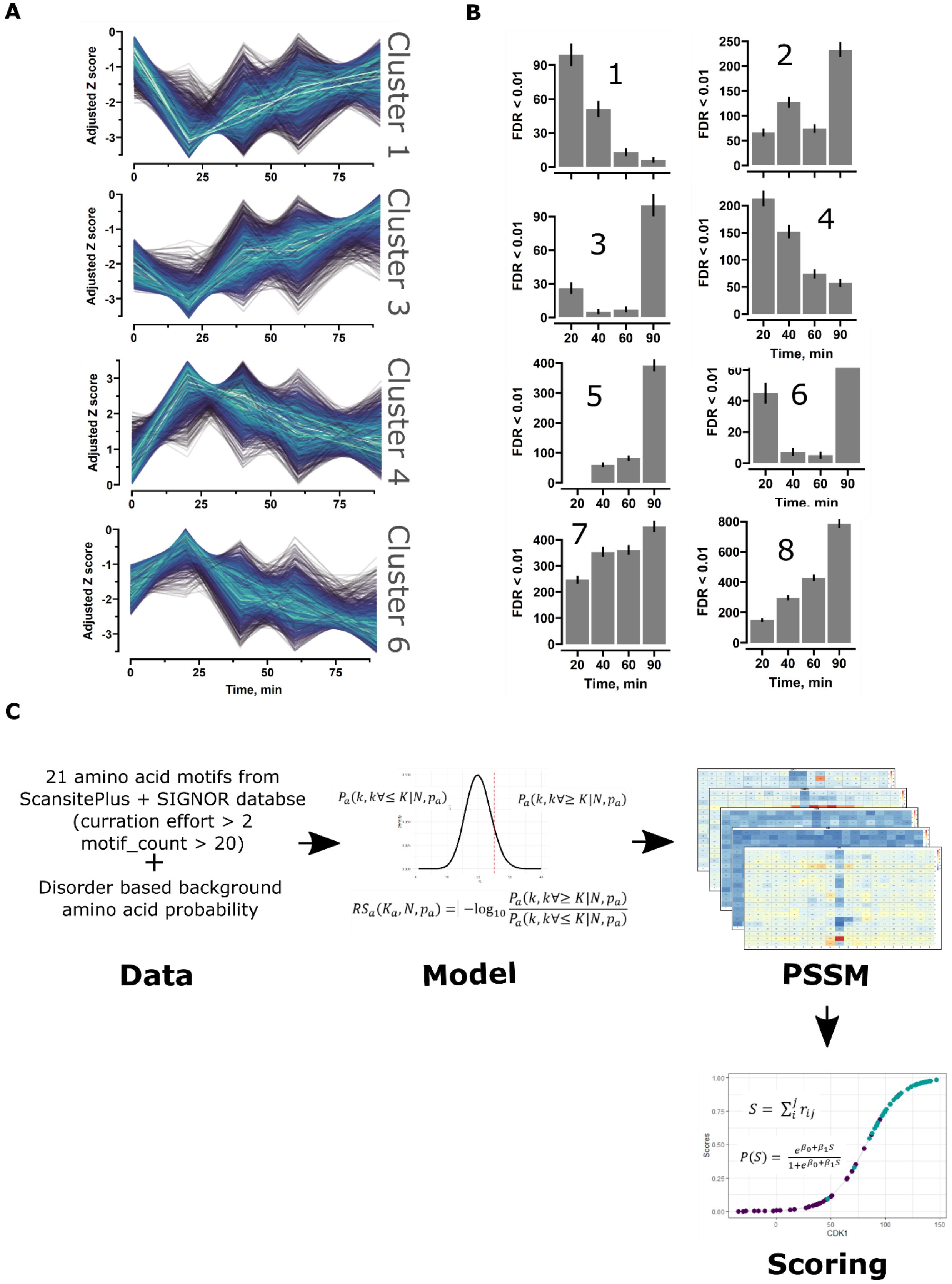
A
Cluster 1
Cluster 3
Cluster 4
Cluster 6
Adjusted Z score
Time, min
B
1
2
3
4
5
6
7
8
FDR < 0.01
Time, min
C
21 amino acid motifs from
ScansitePlus + SIGNOR databse
(curration effort > 2
motif_count > 20)
+
Disorder based background
amino acid probability
Data
$P_a(k, k\forall \leq K|N, p_a)$
$P_a(k, k\forall \geq K|N, p_a)$
$RS_a(K_a, N, p_a) = \left| -\log_{10} \frac{P_a(k, k\forall \geq K|N, p_a)}{P_a(k, k\forall \leq K|N, p_a)} \right|$
Model
PSSM
$S = \sum_i^j r_{ij}$
$P(S) = \frac{e^{\beta_0 + \beta_1 S}}{1 + e^{\beta_0 + \beta_1 S}}$
Scores
CDK1
Scoring

Figure S3. Fuzzy c-means (FMC) clustering and scheme for PSSM generation. Related to Figure 3 and 4. (A) The calculated FCM clusters are shown on the left. Each line corresponds to the dynamics of a single phosphorylation site. The z-scores are plotted on the y axis and time on the y axis. Color indicates the probability of the site for the cluster**. (B)** Bar plots showing the number of significant phosphorylation site alterations (FDR < 0.05) in different clusters at different timepoints after adavosertib treatment. Black bars indicate the squared root of the count. **(C)** Scheme of PSSM based scoring of phosphorylation sites. CDK1 and CDK2 phosphorylation sites were extracted from PSP and SIGNOR databases. The background amino acid frequency was adjusted to 0.4 probability of disorder to account for the tendency of phosphorylation site to lie in disordered protein regions. A binomial probability model was then used to calculate the over or underrepresentation scores for each amino acid at every position generating a 20x21 position specific-scoring matrix (PSSM). Logistic regression model was then trained to transform the arbitrary scores into probabilities.

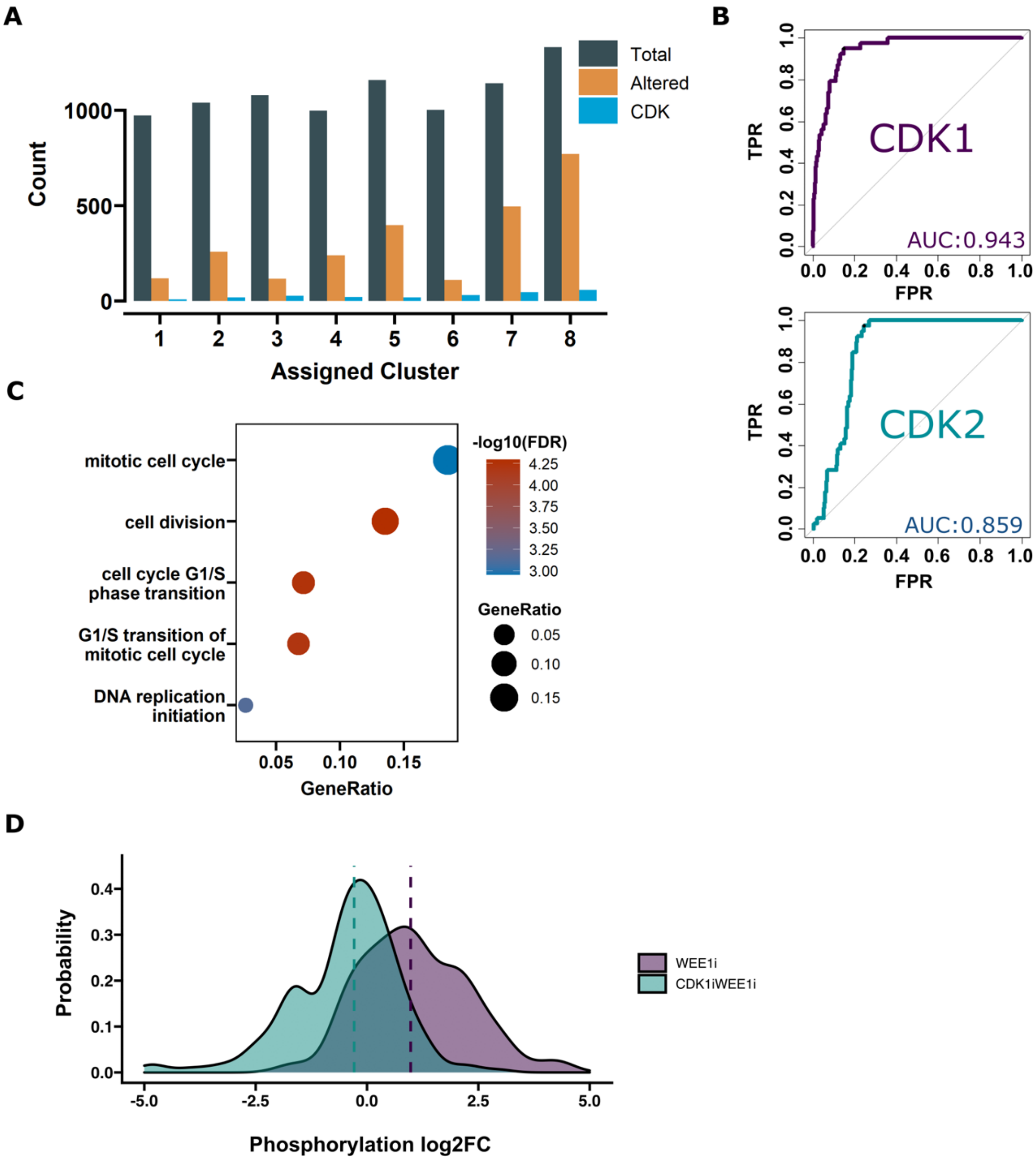

Figure S4. PSSM based prediction of CDK substrates. Related to Figure 4. (A) Bar plot showing count for total, altered an CDK sites in FCM clusters. Total sites represent the total number of phosphorylation stratified to the cluster. Altered represents the number of significantly (FDR < 0.05) altered phosphorylation sites in cluster. CDK represent the number of known CDK sites based on PSP and SIGNOR databases. **(B)** Receiver operating characteristic (ROC) curve for CDK1 and CDK2 phosphorylation site prediction based on PSSM scores. The area under the

curve (AUC) is indicated in the bottom right corner. **(C)** Gene-set overrepresentation analysis for biological processes of predicted CDK1 and CDK2 sites. The size of the dots indicates the number of genes found in the cluster and the color indicates the FDR adjusted p-value. **(D)** Density-plot of log2 transformed fold-changes (log2FC) of phosphorylation site abundances after either adavosertib treated (WEE1i) or adavosertib and CDK1i treated (CDK1iWEE1i) compared to control.

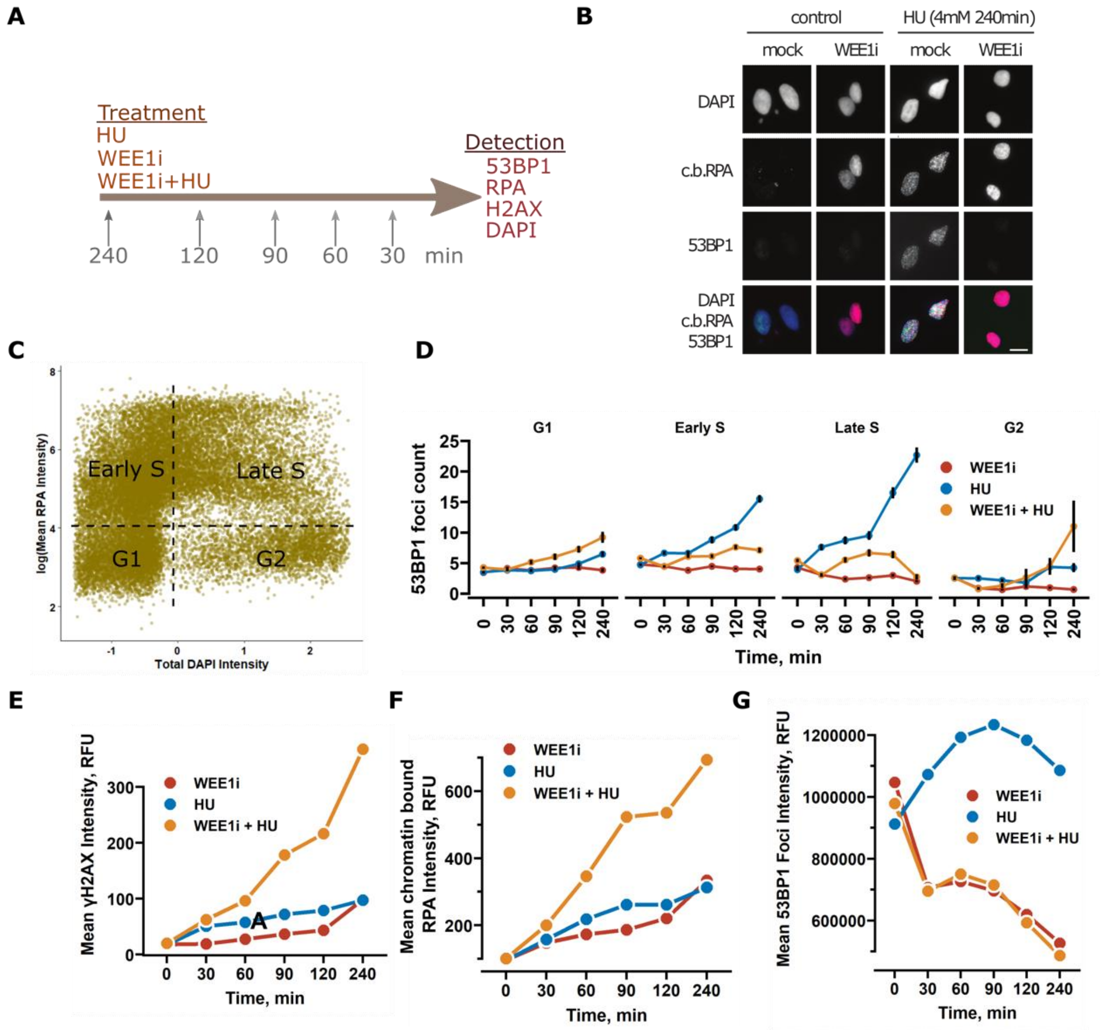

A
Treatment
HU
WEE1i
WEE1i+HU
Detection
53BP1
RPA
H2AX
DAPI
240 120 90 60 30 min
B
control
HU (4mM 240min)
mock
WEE1i
DAPI
c.b.RPA
53BP1
C
Early S
Late S
G1
G2
log(Mean RPA Intensity)
Total DAPI Intensity
D
G1
Early S
Late S
G2
53BP1 foci count
WEE1i
HU
WEE1i + HU
Time, min
E
Mean γH2AX Intensity, RFU
Time, min
F
Mean chromatin bound RPA Intensity, RFU
Time, min
G
Mean 53BP1 Foci Intensity, RFU
Time, min

Figure S5. Cell cycle segmentation and DNA damage marker quantification by QIBC after adavosertib HU treatment. Related to Figure 7. (A) Experimental set-up schematics for adavosertib and HU (4mM) treatments. **(B)** Representative immunofluorescent images of chromatin bound RPA (c.b.RPA), 53BP1 foci of adavosertib and HU treated cells. Scale bar represents 20 μm. **(C)** Cell cycle segmentation with manually set thresholds (dashed black lines) based on the cell cycle profile. The cell cycle segments are indicated in the plot (G1, early S, late S and G2/M). **(D)** Line plots showing mean 53BP1 foci counts at different time points in segmented cell populations with adavosertib and HU treatment. N=2, n>900; black bars indicate SE. **(E)** Mean γH2AX intensity. **(F)** Mean chromatin bound RPA intensity. **(G)** Mean 53BP1 foci intensity. N=2, n>900. N indicates the number of times the experiment has been replicated and n notes the minimal amount of cells quantified per condition.

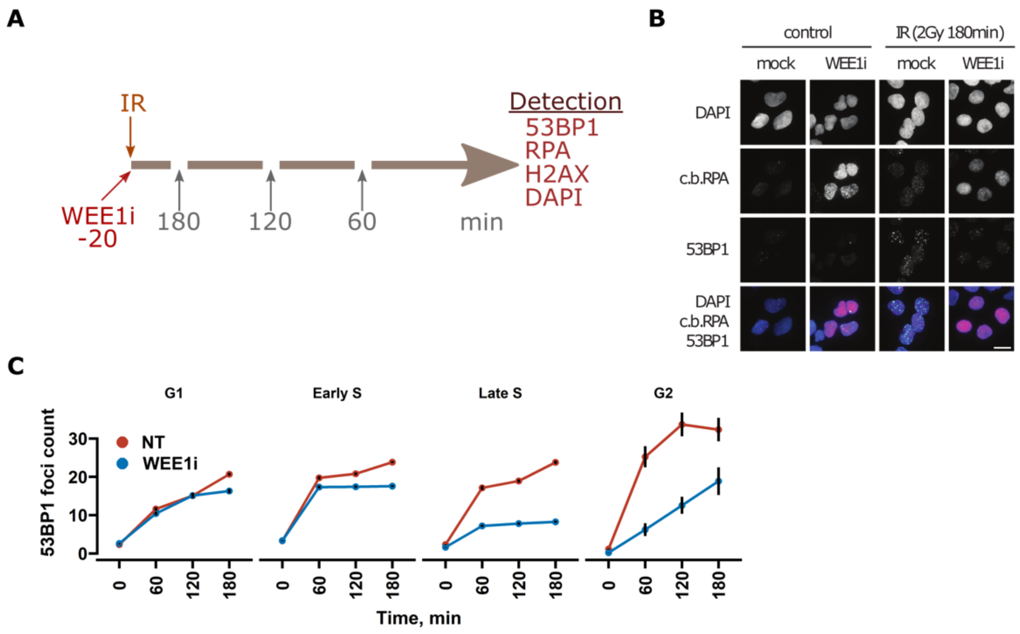

Figure S6. Cell cycle segmentation and DNA damage marker quantification by QIBC after adavosertib IR treatment. Related to Figure 7. (A) Experimental set-up schematics for adavosertib and IR (2 Gy) treatments. WEE1 inhibitor (1 µM) was added 20 min prior to the irradiation (IR). (**B)** Representative immunofluorescent images of chromatin bound RPA (c.b.RPA), 53BP1 foci of adavosertib and HU treated cells. Scale bar represents 20 µm. **(C)** Line plots showing mean 53BP1 foci counts at different time points in segmented cell populations for adavosertib and IR treated cells. All experiments were carried out in triplicate and one representative replicate is shown. N=3, n>1000. N indicates the number of times the experiment has been replicated and n notes the minimal amount of cells quantified per condition. Black bars indicate SE.

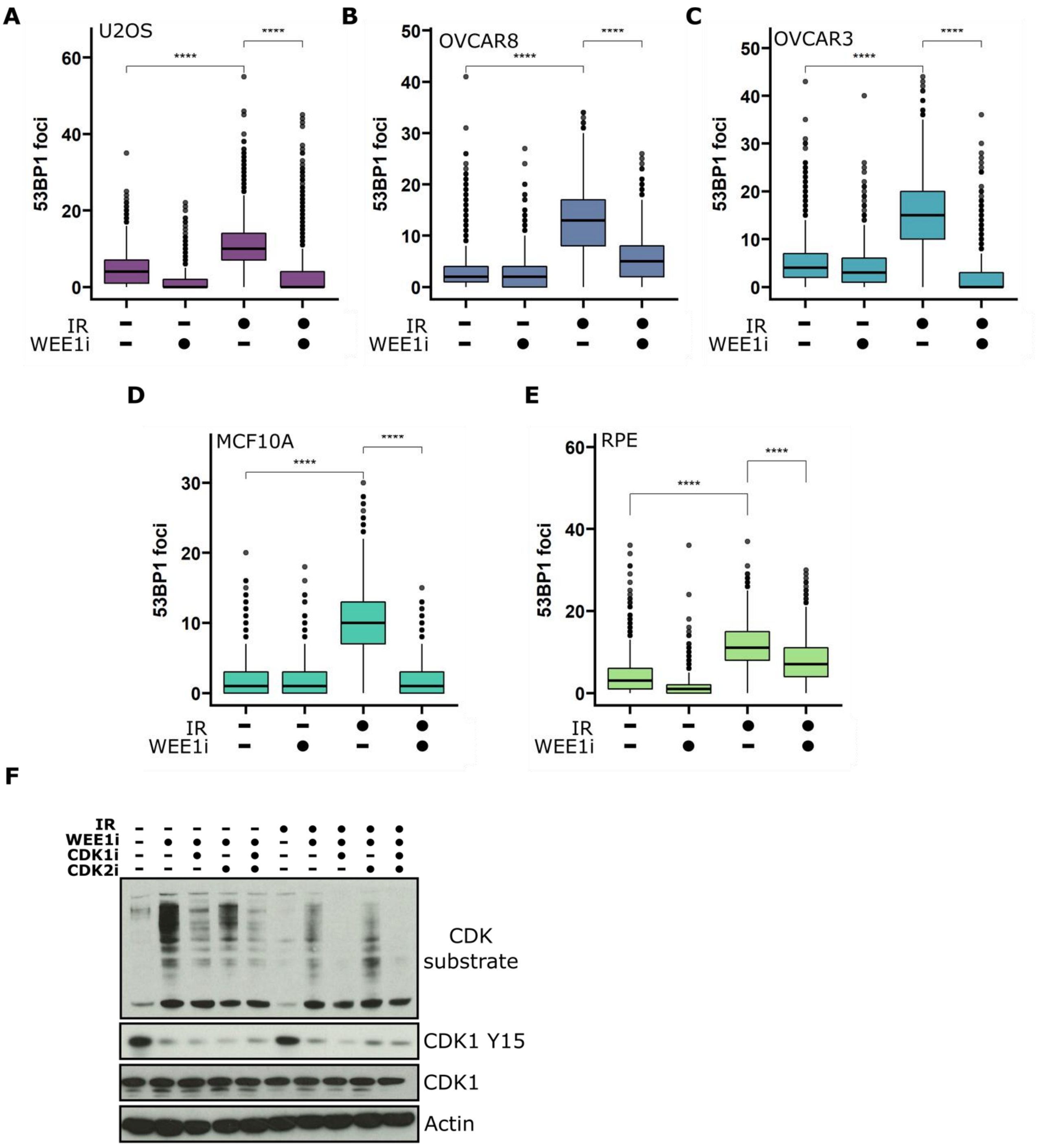
A
U2OS
53BP1 foci
0
20
40
60
****
****
IR
WEE1i
B
OVCAR8
53BP1 foci
0
10
20
30
40
50
****
****
IR
WEE1i
C
OVCAR3
53BP1 foci
0
10
20
30
40
50
****
****
IR
WEE1i
D
MCF10A
53BP1 foci
0
10
20
30
****
****
IR
WEE1i
E
RPE
53BP1 foci
0
20
40
60
****
****
IR
WEE1i
F
IR
WEE1i
CDK1i
CDK2i
CDK substrate
CDK1 Y15
CDK1
Actin

A

Figure S7 WEE1 inhibition impacts 53BP1 in multiple cell lines. Related to Figure 7. (A-E) Boxplot of 53BP1 foci in different cell lines. **(A)** U2OS **(B)** OVCAR8 **(C)** OVCAR3 **(D)** MFC10A **(E)** RPE. The cells were treated with 2 Gy of IR radiation without or with WEE1 inhibition and fixed after 4h. P-values were calculated with t.test. **** indicates p-val < 0.0001. **(F)** Immunoblot of whole cell extracts treated with different inhibitors 3h post IR. Antibodies against total CDK substrate, CDKp Y15, CDK1 and actin were used. All QIBC experiments were carried out at least in duplicate and one representative replicate is shown. N=2, n>500. N indicates the number of times the experiment has been replicated and n notes the minimal amount of cells quantified per condition.
